# A Key Commitment Step in Erythropoiesis Is Synchronized with the Cell Cycle Clock through Mutual Inhibition between PU.1 and S-Phase Progression

**DOI:** 10.1371/journal.pbio.1000484

**Published:** 2010-09-21

**Authors:** Ramona Pop, Jeffrey R. Shearstone, Qichang Shen, Ying Liu, Kelly Hallstrom, Miroslav Koulnis, Joost Gribnau, Merav Socolovsky

**Affiliations:** 1 Department of Pediatrics and Department of Cancer Biology, University of Massachusetts Medical School, Worcester, Massachusetts, United States of America; 2 Department of Reproduction and Development, Erasmus MC, University Medical Center, Rotterdam, The Netherlands; Baylor College of Medicine, United States of America

## Abstract

Hematopoietic progenitors undergo differentiation while navigating several cell division cycles, but it is unknown whether these two processes are coupled. We addressed this question by studying erythropoiesis in mouse fetal liver in vivo. We found that the initial upregulation of cell surface CD71 identifies developmentally matched erythroblasts that are tightly synchronized in S-phase. We show that DNA replication within this but not subsequent cycles is required for a differentiation switch comprising rapid and simultaneous committal transitions whose precise timing was previously unknown. These include the onset of erythropoietin dependence, activation of the erythroid master transcriptional regulator GATA-1, and a switch to an active chromatin conformation at the β-globin locus. Specifically, S-phase progression is required for the formation of DNase I hypersensitive sites and for DNA demethylation at this locus. Mechanistically, we show that S-phase progression during this key committal step is dependent on downregulation of the cyclin-dependent kinase p57^KIP2^ and in turn causes the downregulation of PU.1, an antagonist of GATA-1 function. These findings therefore highlight a novel role for a cyclin-dependent kinase inhibitor in differentiation, distinct to their known function in cell cycle exit. Furthermore, we show that a novel, mutual inhibition between PU.1 expression and S-phase progression provides a “synchromesh” mechanism that “locks” the erythroid differentiation program to the cell cycle clock, ensuring precise coordination of critical differentiation events.

## Abstract 2

During red blood cell development, differentiation and cell cycle progression are intimately and uniquely linked through interdependent mechanisms involving the erythroid transcriptional suppressor PU.1 and the cyclin-dependent kinase inhibitor p57^KIP2^.

## Author Summary

Hematopoietic progenitors that give rise to mature blood cell types execute simultaneous programs of differentiation and proliferation. One well-established link between the cell cycle and differentiation programs takes place at the end of terminal differentiation, when cell cycle exit is brought about by the induction of cyclin -dependent kinase inhibitors. It is unknown, however, whether the cell cycle and differentiation programs are coordinated prior to cell cycle exit. Here, we identify a novel and unique link between the cell cycle clock and the erythroid (red blood cell) differentiation program that takes place several cell division cycles prior to cell cycle exit. It differs from the established link in several respects. First, it takes place at the onset, rather than at the end, of erythroid terminal differentiation, preceding the chromatin changes that enable induction of red cell genes. Second, it is initiated by the suppression, rather than the induction, of a cyclin -dependent kinase inhibitor. It therefore causes the cell to enter S-phase, rather than exit the cell cycle. Specifically, we found that there is an absolute interdependence between S-phase progression at this time in differentiation, and a key commitment step in which, within a short few hours, cells become dependent on the hormone erythropoietin, undergo activating changes in chromatin of red cell genes, and activate GATA-1, the erythroid master transcriptional regulator. Arresting S-phase progression at this time prevents execution of this commitment step and subsequent induction of red cell genes; conversely, arresting differentiation prevents S-phase progression. However, once cells have undergone this key commitment step, there is no longer an interdependence between S-phase progression and the induction of erythroid genes. We identified two regulators that control a “synchromesh” mechanism ensuring the precise locking of the cell cycle clock to the erythroid differentiation program during this key commitment step.

## Introduction

Hematopoietic progenitors execute a cell division program in parallel with a differentiation program in which lineage choice is followed by lineage-specific gene expression. In many differentiation models, cell cycle exit, driven by cyclin-dependent kinase inhibitors (CDKI), is a prerequisite for terminal differentiation, establishing a key interaction between the cell cycle and differentiation programs [1]–[3]. However, it is unclear how the cell cycle and differentiation programs might be linked prior to cell cycle exit. Such links are presumably required to ensure the correct number of differentiated progeny. In addition, it has been speculated that the reconfiguration of chromatin at sites of lineage-specific genes, a necessary step preceding lineage-specific gene expression, may be innately dependent on DNA replication [4],[5]. An intriguing possibility is that the clockwork-like mechanisms regulating orderly cell cycle transitions may also be used, in the context of differentiating cells, to coordinate key steps in differentiation.

Here we studied differentiation of the enucleated red blood cell lineage, which first arises from hematopoietic stem cells in the fetal liver on embryonic day 11 (E11). It replaces a transient, nucleated yolk-sac erythrocyte lineage and persists throughout life. Although many of the committal events that lead to the erythroid phenotype are known, their precise timing in erythroid differentiation, and the manner in which they are coordinated with each other and/or with the cell cycle machinery, is poorly understood. Thus, survival of erythroid progenitors requires both the hormone erythropoietin (Epo), and its receptor, EpoR, a class I cytokine receptor expressed by erythroid progenitors [6]. However, the precise time in erythroid differentiation when progenitors become dependent on Epo had not been defined. The master transcriptional regulator GATA-1 is responsible for the erythroid gene expression profile, in combination with a number of additional transcriptional regulators, including FOG-1, EKLF, SCL/Tal-1, LMO2, Ldb1, E2A, and Zbtb7a [7]–[10]. Though GATA-1 functional activation must precede erythroid gene induction, its precise timing in primary differentiating progenitors is not known. GATA-1 functions are antagonized by PU.1, an Ets transcription factor that acts as a master regulator in the myeloid and B-cell lineages. The mutual inhibition between PU.1 and GATA-1 is thought to underlie cell fate choice in multipotential progenitors [11]–[14]. PU.1 has been implicated in erythroleukemia [13],[15], but its physiological function in erythropoiesis is not known.

Erythroid gene induction by GATA-1 requires an “open chromatin” conformation in the vicinity of erythroid-specific genes. The erythroid-specific β-globin locus is one of the best studied models of lineage-specific gene expression [16],[17]. The active locus is characterized by early replication during S-phase, higher sensitivity to DNase I digestion, low levels of DNA methylation, and post-translational histone tail modifications associated with actively transcribed genes. Conversely, the same locus in non-erythroid cells is DNase I resistant, replicates late in S-phase, and contains histone tail modifications characteristic of silent chromatin. In spite of the detailed knowledge contrasting chromatin states in erythroid cells with non-erythroid cells, the precise time during erythroid differentiation when chromatin reconfiguration occurs is not known. Furthermore, it is not known whether this reconfiguration involves a number of sequential stepwise alterations occurring over a number of cell cycles/differentiation stages or whether the many changes entailed in chromatin activation occur simultaneously.

Here we studied erythroid differentiation using a flow-cytometric assay that identifies sequential stages in erythroid differentiation directly within primary hematopoietic tissue. We found that in mouse fetal liver in vivo, upregulation of CD71 marks cells that are synchronized in S-phase of a single cell cycle, corresponding to the last generation of erythroid colony-forming cells, approximately three cell cycles prior to terminal cell cycle exit. A number of differentiation milestones, whose precise timing in erythroid development was previously unknown, occur during early S-phase of this cycle. These include the onset of Epo dependence, activation of GATA-1 function, and the opening up of chromatin at the β-globin locus. We show that S-phase progression during this specific cell cycle is dependent on downregulation of p57^KIP2^ and is required for execution of these differentiation milestones, including the reconfiguration of chromatin at the β-globin locus. Further, this S-phase dependent rapid differentiation transition is regulated by PU.1 through a newly identified, mutual antagonism between S-phase progression and PU.1 expression that coordinates the precise locking of the differentiation program to the cell cycle clock as cells enter a terminal differentiation phase.

## Results

### Upregulation of Cell-Surface CD71 Marks the Onset of EpoR Dependence in Erythroid Progenitors

**Figure 1:**
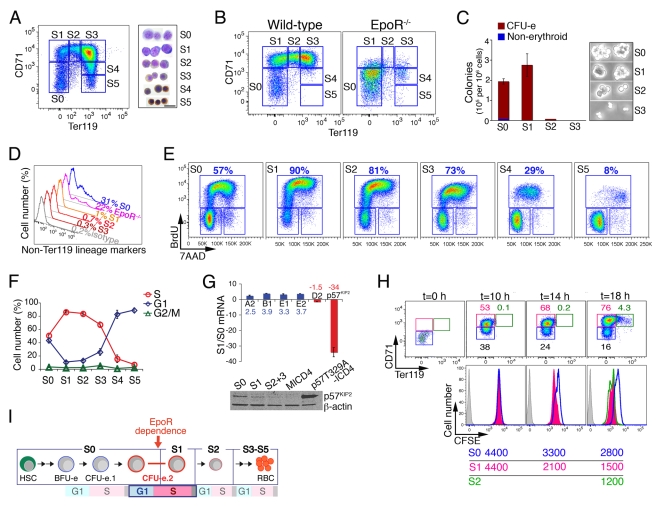
Upregulation of CD71 coincides with the onset of EpoR dependence and with S-phase of the last generation of CFU-e. (A) Fetal liver subsets S0 to S5 form an erythroid developmental sequence. Freshly isolated E14.5 fetal liver was mechanically dissociated and labeled for cell-surface CD71 and Ter119. Cytospin preparations from each subset (right panel) were stained with Giemsa and diaminobenzidine. Scale bar = 20 µ (B) CD71/Ter119 profiles for E12.5 EpoR^−/−^ and wild-type littermate fetal livers. Erythroid differentiation of EpoR^−/−^ cells is blocked at the transition from S0 to S1. Ter119^+^ cells in EpoR^−/−^ liver are nucleated yolk-sac erythrocytes (Figure S1A). Representative of more than four experiments. (C) Erythroid and non-erythroid colony forming potential of fetal liver subsets. 100,000 cells sorted from each of S0 to S3 were plated in methylcellulose in the presence of Epo, IL-3, SCF, and IL-6. Colonies were scored on days 3 (CFU-e) and 10 (CFU-GM). Data are mean ± SE of three independent experiments. Pictures of colonies on day 2 are shown (right panel, lens magnification ×20 for all subsets). There was no statistically significant difference between S0 and S1 (*p* = 0.2, paired *t* test). (D) Non-erythroid lineage-marker expression in S0 to S5. Wild-type or EpoR^−/−^ fetal livers were labeled with CD71 and Ter119 to identify subsets S0 to S5, and with a cocktail of non erythroid lineage markers containing Mac-1, Gr-1, CD41, B220, and CD3, or with isotype control antibody. See also Figure S1B. (E) Representative cell cycle analysis for S0 to S5. Pregnant mice were injected with a pulse of BrdU, and fetal livers were harvested 30 to 50 min post-injection. Cells from each of subsets S0 to S5 were sorted by flow-cytometry and labeled for BrdU incorporation and DNA content (7AAD). (F) Summary of six independent cell cycle analysis experiments as described in (E). Data are mean ± SE. The difference between the number of S-phase cells in S0 and S1 is significant at *p*<0.0001 (paired *t* test). (G) Upper panel: Quantitative RT-PCR analysis of mRNA expression for cyclins A2, B1, E1, E2, D2, and for p57^KIP2^. Data (mean ± SE of three experiments) were normalized to β-actin mRNA in each sample and expressed as S1/S0 fold change (no change = 0). Fold changes are indicated. Lower panel: p57^KIP2^ protein in sorted S0, S1, and S2+3 subsets. Quantitative western blotting with antibodies directed at p57^KIP2^ and β-actin; Near infra-red (NIR) fluorescence–conjugated secondary antibodies. S0 cell transduced with retroviral vector encoding either p57T329A-ICD4 or empty vector (MICD4) were harvested 24 h post-infection and used as positive and negative controls, respectively. (H) CFSE cell tracking of S0 cells as they transition into S1 and S2 in vitro. Sorted S0 cells were pulsed with CFSE and incubated in Epo for 18 h. The time points examined during in vitro incubation are indicated. Upper panel: CD71/Ter119 profiles. The S0 (blue), S1 (red), and S2 (green) gates are indicated with the percentage of cells in each gate. Middle panel: Corresponding CFSE histograms for cells in each of the S0 (blue), S1 (red), and S2 (green) subsets. Lower panel: Median fluorescence intensity of CSFE for the corresponding histograms and colors shown in the middle panel. Representative of 4 similar experiments. (I) Representation of the transition from S0 to S1. S0 contains several CFU-e generations. The last generation of CFU-e, noted as “CFU-e.2,” arises in S0 as an EpoR-independent cell. The onset of EpoR dependence and upregulation of CD71 ( =  transition to S1) occur during S-phase of this cell generation. Upregulation of Ter119 ( =  transition into S2) occurs as the CFU-e.2 cell divides, giving rise to non-CFU-e progeny in S2. Other than the cell cycle corresponding to CFU-e.2, the timing of other cell cycles with respect to differentiation events is not known. See also Figure S1.

Mouse fetal liver between E11 and E15 is primarily an erythropoietic tissue. Cell surface markers CD71 and Ter119 may be used to identify differentiation-stage specific subsets, directly in primary tissue [18]–[20]. Here we divided freshly harvested fetal liver cells into six CD71/Ter119 subsets that we termed S0 to S5 and that form a developmental sequence (Figure 1A). Cells isolated from subsets S1 to S5 show morphological features characteristic of erythroid maturation, including decreasing cell and nuclear size, nuclear condensation, and hemoglobin expression (Figure 1A, right panel). The precise proportion of fetal liver cells within each of the CD71/Ter119 subsets is a function of embryonic age, with the majority of cells being in the early, S0 and S1 subsets in E12. The more mature, S3 to S5 subsets are gradually populated with cells during subsequent embryonic days (E13 to E15) [20].

The EpoR^−/−^ fetal liver is small and lacks morphologically identifiable hemoglobinized erythroblasts of the enucleated (definitive) lineage [6]. Here we found that EpoR^−/−^ fetal liver does not contain subsets S1 to S5 (Figure 1B). This suggested that in the definitive erythropoietic lineage that gives rise to adult-type enucleated red cells, EpoR becomes essential on or prior to the transition from S0 to S1; subsets S1 to S5 are composed almost entirely of Epo-dependent erythroblasts. Of note, the small number (≈5%) of Ter119^+^ cells in the EpoR^−/−^ fetal liver are all nucleated erythrocytes of the transient yolk-sac (primitive) lineage (Figure S1A).

### The Majority of S0 Cells Are Erythroid Progenitors at the CFU-e Stage

Erythroid progenitors have traditionally been identified by their in vitro colony-forming potential. “Colony forming unit-erythroid” (CFU-e) are defined as cells that give rise to colonies containing 8 to 32 hemoglobinized cells after 2–3 days of in vitro culture in Epo [21]. We investigated the colony-forming potential of cells sorted from each of the S0 to S3 subsets (Figure 1C). CFU-e potential was exclusive to S0 and S1 and was lost with the transition to S2. Cells in S2 and S3 gave rise to small, 2 to 4 cell clusters (Figure 1C, right panel).

The frequency of CFU-e obtained from sorted S0 cells was 65%–70% of the frequency from sorted S1 (Figure 1C). S1 consists entirely of Epo-dependent cells of similar maturation, with CFU-e potential (Figure 1A–C). Assuming similar plating efficiency for sorted S0 and S1 (of ≈30%, Figure 1C), this suggested that CFU-e make up 65%–70% of the S0 subset. This is in agreement with our finding that fetal liver cells expressing non-erythroid lineage markers, which were limited to S0, formed up to 30% of this subset (Figure 1D, Figure S1B). Non-erythroid colony-forming progenitors were also restricted to S0, where they formed less than 5% of all colony-forming cells (Figure 1C). Our conclusion that 65%–70% of S0 cells are CFU-e was further supported by single cell RT-PCR, which showed that 68% of S0 cells expressed EpoR mRNA (Figure S1C).

In all the experiments that follow, “S0” refers to S0 cells from which cells expressing non-erythroid markers were excluded by flow-cytometric gating or sorting.

### S1 Cells Are Synchronized in S-Phase of a Single Cell Cycle

To examine the cell cycle status of erythroid subsets S0 to S5 in vivo, we injected pregnant female mice with the nucleotide analogue bromodeoxyuridine (BrdU) and harvested fetal livers 30 min post-injection. We sorted cells from each of S0 to S5 and stained them with antibodies directed at BrdU (Figure 1E,F). Cells that incorporated BrdU were in S-phase of the cell cycle at the time of harvesting. Subsets S4 to S5 showed a rapid decline in the number of S-phase cells, consistent with cell cycle exit of terminally differentiating cells. Unexpectedly, we noted that ≈90% of S1 cells were in S phase, as compared with ≈50% of cells in S0 (Figure 1E,F). In addition, the intensity of the BrdU fluorescence within S1 cells was approximately 50% higher than in S0, suggesting a higher rate of DNA synthesis (Figure S1D). Similar experiments with EpoR^−/−^ fetal liver showed that EpoR appears to have no effect on progenitor cell cycle status (Figure S1E).

Consistent with the higher number of S-phase cells in S1, we found a corresponding increase in the E cyclins in S1 compared with S0 (Figure 1G). Strikingly, we noted >30-fold decrease in the CDKI p57^KIP2^ mRNA, but no significant change in the mRNA of other members of the CIP/KIP CDKI family; there was induction in p27^KIP1^ later in differentiation, in subsets S2 and S3 (Figure 1G and Figure S1F) [22],[23]. The p57^KIP2^ protein also decreased at the S0 to S1 transition (Figure 1G lower panel).

The finding that nearly all S1 cells were in S-phase could be due to an unusual cell division cycle with short or no gap phases. Alternatively, S1 cells may be synchronized in S-phase of the cycle. The latter explanation would require that cells spend only a brief period of a few hours in S1, lasting through part or all of a single S phase. The preceding G1 phase of this same cell cycle would have occurred prior to the transition from S0 to S1. The G2 and M phases of this same cycle would occur as cells upregulate Ter119 and transition into S2.

To investigate these possibilities, we isolated S0 cells by flow-cytometry, labeled them with the cell-tracking dye carboxyfluorescein diacetate succinimidyl ester (CFSE), and followed their Epo-dependent differentiation into S1 in vitro (Figure 1H). By 10 h, 53% of S0 cells transitioned into S1 in the absence of cell division, as indicated by a single CFSE peak for S1 (solid red histogram, t = 10 h) that was identical in intensity to that of the CFSE peak for S0 (blue histogram, t = 10 h; median CFSE fluorescence for both S1 and S0 peaks = 4,400). This suggested that the transition from S0 to S1 occurred in the absence of cell division, within a single cell cycle. Four hours later, at t = 14 h, essentially all S1 cells had divided once, as indicated by the halving of the CFSE signal (red histogram at t = 14 h, CFSE fluorescence = 2,100). The simultaneous division of S1 cells suggested they were synchronized in their cell cycle phase. By contrast, only a portion of S0 cells, which were presumably asynchronous in their cell cycle phase, had divided at this time, resulting in a biphasic CFSE peak (blue histogram, t = 14 h).

Taken together, these results suggest that the most mature CFU-e progenitor (“CFU-e.2”, Figure 1I), capable of giving rise to an eight-cell colony, traverses S0, S1, and enters S2 within a single cell cycle. This progenitor arises in S0, becomes Epo dependent, and upregulates CD71, transitioning into S1 during S-phase of its cell cycle. Upregulation of Ter119 occurs at approximately the same time that it completes its cycle and divides, giving rise to progeny that lack CFU-e activity in S2 (Figure 1C). These conclusions are consistent with essentially all S1 cells being in S-phase (Figure 1E,F), and with our finding that nearly all S1 cells are sensitive to hydroxyurea, a drug that specifically targets S-phase cells (Figure S2A). These conclusions are consistent with a number of other observations: the loss of CFU-e activity with Ter119 expression (Figure 1C, [24]), the short time span (<15 h) that freshly sorted S0 cells require to transition through S1 and into S2 (compare with an estimated cell cycle length of 16 h for a CFU-e cell that will undergo three cell divisions in 48 h, giving rise to an eight cell colony), and with early work suggesting that Epo dependence first occurs in early S-phase of a specific CFU-e cell generation [25]. These conclusions are also consistent with the finding that EpoR^−/−^ embryos have normal numbers of CFU-e [6]: though EpoR^−/−^ embryos lack S1 cells, all the CFU-e in S1 first arise as Epo-independent cells in S0, where they are presumably retained in the EpoR^−/−^ fetal liver.

### S-Phase Progression Is Required for the Transition from S0 to S1

**Figure 2:**
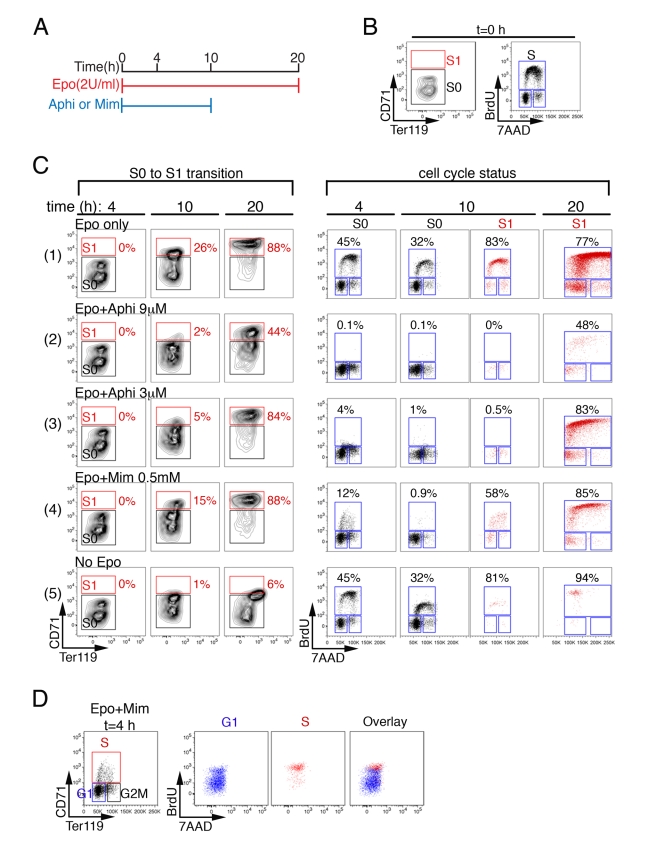
The S0 to S1 transition requires S-phase progression. (A) Design of experiments illustrated in sections (B–D). Flow-cytometrically sorted S0 cells were incubated in Epo for 20 h. In the first 10 h, cells were also in the presence or absence of a cell cycle blocking drug, either aphidicolin (Aphi) or mimosine (Mim). DMSO was added to control cells in the Aphi experiments. Aphi or Mim were removed by washing at t = 10 h. Cell cycle status and CD71/Ter119 expression were examined at 4, 10, and 20 h. (B) CD71/Ter119 expression and BrdU/7AAD cell cycle profile of freshly sorted S0 cells at t = 0. Cells were incubated in the presence of BrdU for 30 min prior to fixation, permeabilization, and staining with antibodies for CD71, Ter119, and BrdU. (C) CD71/Ter119 expression (left columns) and corresponding cell cycle profile of S0 and S1 cells (right columns) at the indicated time points. The presence or absence of Epo, Aphi, or Mim is indicated above each row of histograms. The indicated percentages correspond to the fraction of cells in S1 (left columns) or in S-phase of the cycle (right columns). Note that Ter119 signal is reduced in fixed and permeabilized cells compared with equivalent non-fixed cells (e.g., Figure 1H and Figure S2A), since the Ter119 epitope is partially detergent soluble. Data representative of five experiments. (D) “Back-gating” analysis for CD71 expression of S0 cells that are either in G1 (blue) or in S-phase (red), at t = 4 h in the presence of Epo+Mim. The same BrdU/7AAD profile as in section (C), row 4, t = 4 h. See also Figure S2.

There are two ways to explain how upregulation of CD71, a differentiation event, might coincide with S-phase, a cell cycle event. These events may have each been initiated in parallel by a common upstream regulator, such as the EpoR, since both occur at the time that cells become EpoR dependent. Alternatively, there may be a direct mechanistic link between the differentiation and cell cycle programs. To distinguish these possibilities, we examined whether a block to S-phase progression would interfere with CD71 upregulation (Figure 2). We incubated sorted S0 cells in vitro for 10 h in the presence of Epo, and either in the presence or absence of aphidicolin, an inhibitor of DNA polymerase that arrests S-phase progression [26]. At t = 10 h, cells were washed free of aphidicolin and incubated in Epo alone for an additional 10 h (Figure 2A). In the initial 10 h of incubation, there was an Epo-dependent transition of cells from S0 to S1 (Figure 2C, rows 1 and 5). However, the presence of aphidicolin blocked this transition (Figure 2C, rows 2 & 3, t = 10 h). Both S-phase and the transition into S1 resumed once the cells were washed free of aphidicolin (Figure 2C, rows 2 & 3, t = 20 h). These observations suggested that the transition from S0 to S1 occurred during S-phase and required both Epo and S-phase progression.

We also examined the effect of mimosine, a plant amino acid that blocks cell cycle progression in late G1 [27]. We incubated sorted S0 cells in Epo and in the presence or absence of mimosine. By 4 h of incubation, the majority of cells were arrested in G1. However, a small fraction of cells (12%) could be seen in S-phase at t = 4 h (Figure 2C, row 4, BrdU/7AAD at t = 4 h). Presumably, at the time mimosine was added, these cells were advanced in their cell cycle beyond the point at which mimosine exerts its block. BrdU/7AAD analysis showed that these cells were in the early half of S-phase and expressed the highest CD71 levels within the S0 subset (Figure 2D, cells marked in red). By t = 10 h, no S-phase cells were seen in S0, presumably because they have now transitioned into S1, where a similar number of cells (15%) had newly appeared (Figure 2C, row 4, BrdU/7AAD for S0 at t = 10 h, and CD71/Ter119 for S1 at t = 10 h). These observations were consistent with the onset of CD71 upregulation occurring in early S-phase in S0, culminating in the transition to S1 later within that same S-phase.

CD71, the transferrin receptor, is required during erythroid differentiation in order to facilitate cellular uptake of iron for hemoglobin synthesis. CD71 is also expressed, albeit at lower levels, on all cycling cells. We therefore examined whether, in the context of S1 cells, CD71 might be required specifically for S-phase progression. We used RNAi to prevent CD71 upregulation in S0 cells during their incubation in Epo (Figure S2B,C). The failure of these cells to upregulate CD71 did not interfere with the number of cells in S-phase (Figure S2B). Therefore, the link between S-phase progression and CD71 upregulation in S1 cells is not due to a cell cycle function for this gene.

### The S0 to S1 Transition Is Marked by Downregulation of PU.1 and GATA-2 and Precedes Induction of Erythroid-Specific Genes

**Figure 3:**
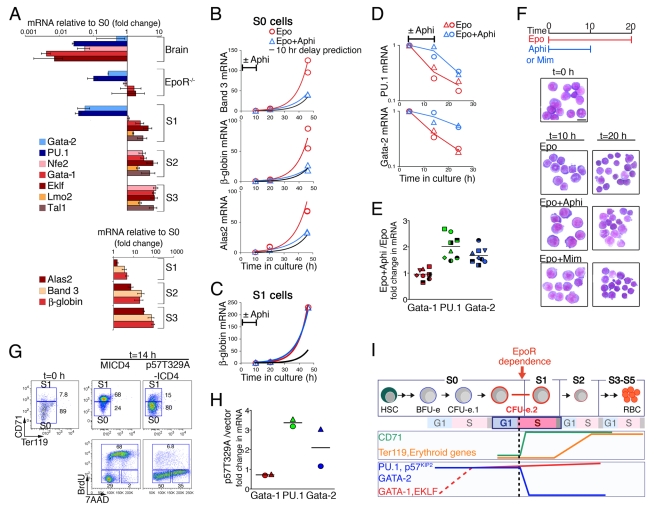
Block of S-phase progression at the S0 to S1 transition arrests the erythroid differentiation program. (A) Expression of transcriptional regulators (upper panel) and erythroid-specific genes (lower panel) in sorted fetal liver subsets S0 to S3, in fetal brain, and in lineage marker-depleted EpoR^−/−^ fetal liver. mRNA measured by quantitative RT-PCR, normalized to the β-actin mRNA, and expressed as a ratio to the S0 subset. Data are mean ± SD of 2 (for EpoR^−/−^ and brain) or 3 (for S0 to S3) independent experiments. (B, C) Effect of aphidicolin-mediated S-phase arrest on erythroid-specific gene expression. Sorted S0 (in B) or S1 (in C) cells were incubated in Epo, and in either aphidicolin (3 µM) or DMSO, for the first 10 h. Aphidicolin and DMSO were removed by washing at t = 10 h. mRNA was measured by qRT-PCR, normalized to β-actin, and expressed as a ratio to mRNA at t = 10 h in Epo+DMSO. Duplicate independent experiments shown, fitted with exponential curves. Black curves are the calculated time course for a 10 h delay in induction for each gene, obtained by shifting the respective red curves (describing time course for Epo + DMSO) by 10 h. (D) Effect of aphidicolin-mediated S-phase arrest on PU.1 and GATA-2. Experiment and mRNA measurement as described for (B), except that aphidicolin and/or DMSO were applied at t = 4 h and removed at t = 14 h. Data from two independent experiments. (E) mRNA expression of transcriptional regulators GATA-1, GATA-2, and PU.1 at the end of a 10 h incubation in Epo + aphidicolin, compared with cells incubated in Epo + DMSO. Experimental design and mRNA measurement as described in section (D), with data pooled from nine independent experiments (each with a distinct symbol). Bar indicates position of mean. Differences between Epo+ aphidicolin and Epo control samples are significant (paired two-tailed *t* test) for GATA-2 (*p* = 0.004) and for PU.1 (*p* = 0.002). (F) Effect of aphidicolin or mimosine-mediated S-phase arrest on erythroblast morphology during differentiation of S0 cells in vitro. Sorted S0 were incubated in Epo, and for the first 10 h of incubation, also in the presence of either aphidicolin or mimosine. Control cells were incubated in Epo alone throughout. Cytospin preparations of cells at 10 and 20 h of incubation are shown, stained with Giemsa. Morphological maturation (decreasing cell and nuclear size), compared with control cells, was arrested at t = 10 h, and remained delayed when the block to cell cycle progression was removed (t = 20 h). (G) S-phase arrest by overexpression of a non-degradable mutant of p57^KIP2^ (p57T329A). Sorted S0 cells were transduced with retroviral vector expressing p57T329A linked to IRES-hCD4 reporter, or with control vector (MICD4). Cells were cultured for 15 h in IL-3 and SCF and transferred to an Epo containing medium at t = 0, to allow their transition from S0 to S1. CD71/Ter119 profiles of infected, hCD4 positive S0 at t = 0 and t = 14 h (upper panels) and BrdU/7AAD cell cycle profiles (lower panels) at t = 14 h are shown. Representative of 4 experiments. Transduction efficiency of p57^KIP2^ exceeded 90% in all experiments. (H) Transcriptional regulators GATA-1, GATA-2, and PU.1 in cells expressing p57T329A. Experiment as described in (G). mRNAs measured by qRT-PCR following 24 h of incubation in Epo, and expressed relative to cells transduced with control vector (MICD4) after normalization to β-actin. Duplicate independent experiments are shown. (I) Representation of erythroid gene expression at the transition from S0 to S1. GATA-1 and other activators of the erythroid transcriptional program are induced at an unknown time preceding the S0/S1 boundary, and increase modestly with further differentiation. P57^KIP2^, PU.1, and GATA-2 are expressed in S0 and are markedly downregulated at the S0 to S1 transition. Erythroid specific Ter119, β-globin, ALAS2, and Band3 are induced subsequent to the S0/S1 boundary. S-phase arrest at this stage (dashed black line) results in arrest of all subsequent events, including PU.1 and GATA-2 downregulation, CD71 and Ter119 expression, erythroid-specific gene induction, and morphological maturation. See also Figure S3.

To investigate the link between S-phase and the erythroid differentiation program, we examined expression of erythroid transcriptional regulators and erythroid-specific genes in freshly sorted fetal liver subsets and in fetal brain (Figure 3A). We found that the GATA-1 mRNA was present in S0 cells, at 200-fold higher levels than in fetal brain (Figure 3A) and 40-fold higher level than in Mac-1^+^ cells (Figure S3A). It increased a further ≈2-fold with the transition from S0 into S1 and continued to increase in S2 and S3. Of note, total RNA per cell decreased 4-fold over the course of differentiation from S2 to S4 (Figure S3B), suggesting an overall modest increase in GATA-1 mRNA per cell over this period. Other erythroid transcriptional activators and GATA-1 associated factors, including EKLF, NF-E2 [28], SCL/Tal-1, and Lmo2, showed a similar expression pattern to that of GATA-1 (Figure 3A). Therefore, expression of GATA-1 and of other activators of the erythroid transcriptional program precedes the transition from S0 to S1. By contrast, we found that PU.1, a repressor of GATA-1 function, and GATA-2, a target of GATA-1-mediated repression [29], were both downregulated ≈30-fold and ≈20-fold, respectively, at the S0 to S1 transition, becoming undetectable with further differentiation (Figure 3A). Prior to its downregulation, the level of PU.1 in S0 cells was comparable to that of myeloid Mac-1^+^ cells (Figure S3A). PU.1 protein levels also declined with the transition from S0 to S1 (Figure S3C).

EpoR^−/−^ fetal liver cells, though apparently arrested at the S0 stage (Figure 1B), have a similar expression pattern of transcriptional regulators to wild-type S1 (Figure 3A). Therefore, downregulation of PU.1 and GATA-2 at the S0 to S1 transition, as well as the preceding induction of GATA-1, are independent of EpoR signaling.

We examined expression of several erythroid-specific GATA-1 target genes: β-globin (*Hbb-b1*); the first enzyme of heme synthesis, aminolevulinic acid synthase 2 (ALAS2); and the anion exchanger Band 3 (*Slc4a1*), a major erythrocyte membrane protein [30]. There was a modest increase in their expression at the S0 to S1 transition, followed by a 30–100-fold induction during subsequent differentiation in S2 and S3 (Figure 3A). Expression of the EpoR gene, itself a GATA-1 target, increased 10-fold above its S0 level with the transition to S1 (Figure S3D). Taken together, induction of erythroid GATA-1 target genes and repression of GATA-2 suggest that GATA-1 function is activated at the S0 to S1 transition. The modest increase in GATA-1 mRNA at this time suggests that its activation may be principally a result of PU.1 downregulation.

### S-Phase Arrest at the S0 to S1 Transition Blocks Induction of Erythroid-Specific Genes

We had found that S-phase progression at the transition from S0 to S1 was required for CD71 upregulation (Figure 2). We therefore examined whether S-phase progression at this time was also required for induction of erythroid-specific genes. We cultured sorted S0 cells in Epo for 10 h, a period sufficient for 25%–50% of cells to transition into S1 (Figures 1H, 2C), and examined the effect of adding aphidicolin to the culture. Cells were then washed free of aphidicolin, continuing incubation in Epo alone. Cells incubated in Epo alone for the entire period showed ≈50- to 100-fold induction in the mRNAs for β-globin, Band 3, and ALAS2 (Figure 3B, red curves). By contrast, cells that were subject to aphidicolin treatment during the initial 10 h showed reduced mRNA induction by the end of the culture period (Figure 3B, blue curves). The reduced mRNA levels corresponded closely to the levels predicted had there been a 10 h delay in the time course of induction for each of the genes (Figure 3B, black curves). Therefore, induction of erythroid-specific genes was likely blocked during the incubation period in aphidicolin.

We also examined whether S-phase arrest interferes with erythroid gene induction if applied at the S1 stage of differentiation. We sorted S1 cells and incubated them in Epo, either in the presence or absence of aphidicolin. Unlike S0 cells, aphidicolin-mediated S-phase arrest of S1 did not interfere substantially with their induction of erythroid specific genes, as shown by the unperturbed induction of β-globin, Alas2, and Band 3 (Figure 3C, Figure S3E) or with the upregulation of Ter119 (Figure S3F). Therefore, S-phase progression is required for activation of erythroid-specific genes, specifically at the S0 to S1 transition, but not a few hours later when the cells have traversed into S1. The lack of effect of aphidicolin on mRNA induction in S1 suggests its effects in S0 are not due to non-specific suppression of transcription.

### S-Phase Arrest at the S0 to S1 Transition Blocks Downregulation of PU.1 and GATA-2 and Arrests Erythroid Morphological Maturation

Transcripts for PU.1 and GATA-2 are markedly downregulated at the transition from S0 to S1 (Figure 3A). We examined whether S-phase arrest interferes with their downregulation. Sorted S0 cells were incubated in Epo for 4 h, at which time, just prior to their transition into S1 (Figure 2C), aphidicolin was added to the cultures for a period of 10 h. Cells were then washed free of aphidicolin and incubated in Epo for a further 10 h. Aphidicolin halted the downregulation of both PU.1 and GATA-2, which resumed once the cells were washed free of the drug (Figure 3D,E). Similar results were obtained in cells treated with mimosine (Figure S3G). Therefore, S-phase progression is required for downregulation of PU.1 and GATA-2 at the S0 to S1 transition. Of note, GATA-1, Nfe2, and Lmo2 mRNAs, which did not change significantly during the transition from S0 to S1 (Figure 3A), were not altered significantly by the aphidicolin treatment (Figure 3E, Figure S3H).

We also examined the effects of aphidicolin or mimosine treatment on morphological maturation of S0 cells cultured in Epo. Following 10 h in Epo in the presence of aphidicolin or mimosine, cells appeared larger than cells incubated in Epo alone. This suggested that, while S-phase progression and the erythroid differentiation program had both arrested, cell growth was not perturbed (Figure 3F). Cells were then washed free of aphidicolin or mimosine and cultured in Epo alone. By 20 h, erythroid maturation had resumed in cells that were initially incubated in cell cycle blocking drugs, as judged by decreasing cell size, nuclear condensation, and decreased nuclear to cytoplasmic ratio, but was nevertheless delayed when compared with control cells. These results are consistent with the effect of S-phase arrest on gene expression (Figure 3B,D,E) and suggest that S-phase progression at the S0 to S1 transition is a key requirement for activation of the erythroid differentiation program.

### Preventing p57^KIP2^ Downregulation Blocks S-Phase Progression at the S0 to S1 Transition and Arrests Erythroid Differentiation

Expression of p57^KIP2^ mRNA decreases over 30-fold at the S0 to S1 transition, and this is associated with downregulation of the p57^KIP2^ protein (Figure 1G). To examine the effect of preventing p57^KIP2^ downregulation, we generated a point mutant of p57^KIP2^, p57T329A, analogous to a proteolysis-resistant human p57^KIP2^ mutant [31]. Sorted S0 cells were infected with bicistronic retroviral vectors expressing either wild-type p57^KIP2^ or p57T329A, linked through an internal ribosomal entry site (IRES) to a human CD4 (hCD4) reporter; control cells were infected with retroviral vector expressing the IRES-hCD4 construct only (MICD4). To allow expression of the transduced p57^KIP2^, infected cells were cultured for 15 h in stem-cell factor (SCF) and interleukin 3 (IL-3), cytokines that sustain viability of progenitors but, unlike Epo, do not support differentiation from S0 to S1. Infected S0 cells were then transferred to Epo for 14 h (Figure 3G). Expression of either wild-type (unpublished data) or mutant p57^KIP2^, but not expression of MICD4, resulted in a block to S-phase progression and inhibited the transition from S0 to S1 (Figure 3G). Further, PU.1 mRNA was >3-fold higher in cells expressing p57^KIP2^ compared with control cells expressing vector only (Figure 3H), suggesting that, as in the case of aphidicolin-mediated S-phase arrest, p57^KIP2^-mediated S-phase arrest prevents downregulation of PU.1 at the transition from S0 to S1. Erythroid morphological maturation, but not cell growth, of p57T329A-transduced cells was also arrested (Figure S3I).

Taken together, upregulation of CD71, which defines the transition from S0 to S1, identifies a key differentiation transition within the last generation of CFU-e (“CFU-e.2”, Figure 3I). It marks the onset of EpoR dependence and occurs exclusively during S-phase of the cell cycle. Induction of GATA-1 and other activators of the erythroid transcriptional program precedes this transition, whereas induction of erythroid-specific genes such as β-globin and Ter119 follows it. The S0 to S1 transition coincides with rapid downregulation of p57^KIP2^, PU.1, and GATA-2. Both Epo and S-phase progression are required for upregulation of CD71. S-phase progression at the S0 to S1 transition requires the downregulation of p57^KIP2^ and is in turn required for the downregulation of PU.1 and GATA-2 and the subsequent activation of erythroid-specific genes. By contrast, S-phase arrest in S1 cells does not affect erythroid gene activation (Figures 3C, S3E–F).

### Persistently Elevated PU.1 Arrests S-Phase Progression and Blocks Erythroid Differentiation

**Figure 4:**
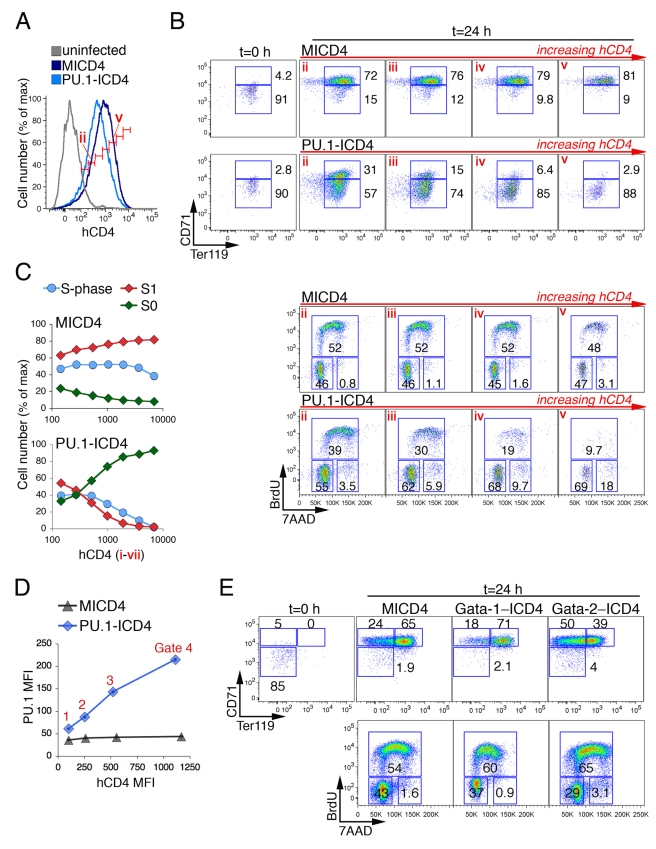
PU.1, but not GATA-2, inhibits the transition from S0 to S1. (A–C) Effect of exogenous PU.1 on the transition from S0 to S1. Sorted S0 cells were transduced with retroviral vector expressing PU.1-IRES-hCD4 (PU.1-ICD4) or control vector (MICD4) and were incubated in IL-3 and SCF for 15 h before being transferred to Epo for 24 h. (A) Expression profiles of the hCD4 reporter in cells transduced with either PU.1-ICD4 or with control MICD4, at 24 h of Epo culture. Vertical, narrow gates each containing cells of relatively uniform hCD4 expression are shown and numbered (i to vii) and are used in the analysis shown in sections (B) and (C) below. (B) CD71/Ter119 (upper panels) and cell cycle (lower panels) analysis of cells in individual hCD4 gates (ii) to (v), at t = 24 h. Higher hCD4 indicates higher PU.1 expression in cells transduced with PU.1-ICD4. (C) Summary of histogram data in (B), correlating cell cycle and differentiation data to hCD4 expression. Each data point corresponds to one of the hCD4 vertical gates marked in (A). Data are representative of five independent experiments. (D) Linear correlation between PU.1 expression levels as measured by flow-cytometry using a PU.1-specific antibody, and hCD4 levels, in cells transduced either with PU.1-ICD4 or with control MICD4. Summary of data shown in Figure S4A,B. Data are representative of 2 independent experiments. (E) Effect of exogenous GATA-1 or GATA-2 on S0 cell differentiation and cell cycle. Experimental design as in section (A–C). CD71/Ter119 and cell cycle profiles are shown for hCD4+ cells transduced with the indicated retrovirus. Flow-cytomeric expression of retroviral constructs is shown in Figure S4C.

Both PU.1 and GATA-2 were rapidly and dramatically downregulated at the transition from S0 to S1 (Figure 3A, Figure S3A). We examined the effect of preventing this downregulation by expressing either PU.1 (Figure 4A–D) or GATA-2 (Figures 4E, S4C,D) in S0 cells using retroviral constructs and a similar strategy to that described above for p57^KIP2^. Following infection, S0 cells were cultured for 15 h in IL-3 and SCF and then transferred to Epo for 24 h, when CD71/Ter119 and cell cycle profiles were examined (Figure 4A–C). We divided the PU.1 expression profile at t = 24 h into 7 sequential hCD4 gates labeled (i) to (vii) (Figure 4A), each containing cells with increasing levels of the hCD4 reporter and, therefore, increasing levels of PU.1. By measuring PU.1 protein directly in fixed and permeabilized cells using a PU.1-specific antibody and flow-cytometry, we found that hCD4 protein expression was a reliable reporter of exogenous PU.1 protein expression in our system (Figures 4D, S4A–B); expression of transduced PU.1 was also measured by qPCR (Figure S4D). Sequential hCD4 gates were also obtained for control cells expressing the empty MICD4 vector. PU.1 expression blocked transition from S0 to S1, with the number of cells transitioning into S1 declining as PU.1 expression increased (Figure 4B, upper panels). PU.1 expression also resulted in a decrease in the number of S-phase cells, with cells arresting principally at the transition from G1 to S-phase, though there was also an increase in the number of cells within G2 or M (Figure 4B, lower panels). The decrease in the number of cells in S1 was paralleled by decreased S-phase cell number, suggesting a direct correlation between the PU.1-mediated block of the transition from S0 to S1, and its inhibitory effect on S-phase (Figure 4C). Therefore, PU.1 inhibits both S-phase and erythroid differentiation at the S0 to S1 transition.

Since the downregulation of both PU.1 and p57^KIP2^ are required for S-phase progression and for the transition from S0 to S1 (Figure 3G, Figure 4B,C), we examined whether PU.1 may be a regulator of p57^KIP2^. However, we found that exogenous expression of PU.1 did not prevent downregulation of p57^KIP2^ (Figure S4E). Therefore, PU.1's inhibitory effect on S-phase is not mediated via p57^KIP2^.

In contrast to PU.1, expression of GATA-2 in S0 cells did not prevent transition into S1, though it somewhat reduced the subsequent transition from S1 to S2 (Figure 4E). GATA-1 overexpression in S0 cells had the opposite effect, of promoting the transition from S1 to S2. There was no significant effect of either GATA-1 or GATA-2 on the cell cycle profile (Figure 4E).

### The S0 to S1 Transition Coincides with a Switch in the Timing of Replication of the β-Globin Locus

**Figure 5:**
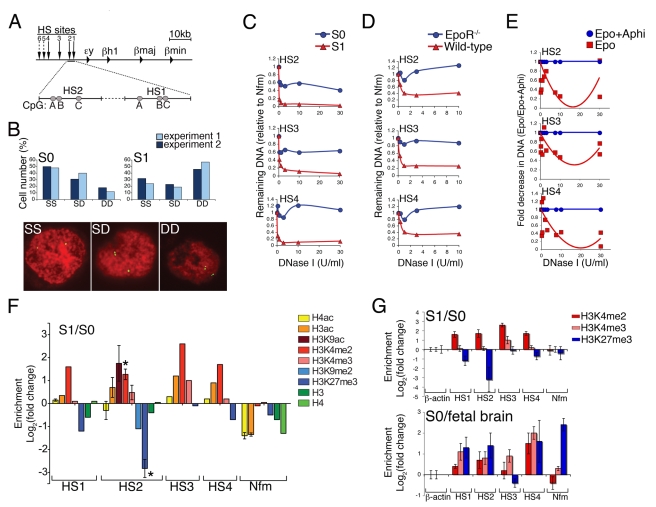
The S0 to S1 transition coincides with an S-phase dependent switch in the state of chromatin at the β-globin locus. (A) A map of the mouse β-globin locus. β-globin genes are indicated with horizontal arrowheads. Vertical arrows indicate DNase I hypersensitivity sites (HSs). Solid arrows for HS1 to HS4 indicate sites examined in experiments below. Expanded HS1 and HS2 sites show locations of CpG dinucleotides (labeled HS1A, B, C, and HS2A, B, C). (B) FISH analysis of the timing of replication at the β-globin locus. Pregnant female mice were injected with BrdU 30 min prior to harvesting of fetal livers. Sorted S0 and S1 cells were fixed and stained for BrdU (red) to identify S-phase cells. Cells were hybridized with a probe to the β-major gene (green), and the number of hybridization spots in 100 BrdU-positive cells in consecutive fields for each of S0 or S1 were counted by fluorescence microscopy, in two independent experiments. Cells were scored as DD (two double dots, indicating both alleles have replicated), SD (one single and one double dot, indicating only one allele has replicated), or SS (two single dots, indicating neither allele has replicated). Examples of nuclei with each of the patterns are shown. (C) DNase I sensitivity in S0 or S1 cells. Nuclei were prepared from sorted S0 and S1 cells and digested with increasing concentrations of DNase I for 10 min. DNA was extracted and quantitative PCR used to measure remaining DNA at each of HS2, HS3, and HS4 using 150 bp amplicons. DNA measurements were normalized to DNA amplified at the neuronal gene Nfm. Representative of three independent experiments. (D) DNase I sensitivity of whole fetal liver from E12.5 EpoR^−/−^ and littermate wild-type controls. Method as in (C). Representative of three independent experiments. (E) Effect of S-phase arrest on development of DNase I hypersensitivity. Sorted S0 cells were incubated in Epo for 10 h to allow transition to S1, in the presence of aphidicolin (Aphi) or DMSO (control). Nuclei were prepared and DNase I sensitivity measured as described in (C). DNA in each sample was normalized to Nfm and expressed as a ratio of DNA in cells incubated in Epo + aphidicolin to DNA in cells incubated in Epo + DMSO. Individual data points are pooled from 3 independent sorting and digestion experiments; curves are fitted second order polynomials. (F,G) ChIP-qPCR in sorted S0 and S1 cells and in fetal brain. ChIP was performed with the indicated antibodies and with control, isotype-matched antibody. qPCR was of 150 bp amplicons at the LCR HSs and at the β-actin and Nfm genes. Data are expressed as enrichment over input DNA in S1 relative to S0 (in F and in G, upper panel) or S0 relative to fetal brain (in G, lower panel). Each sample was normalized to β-actin after subtraction of background (ChIP background was the signal with isotype control antibodies, which was <10% of the signal obtained with specific antibodies). (F) Summary of seven independent ChIP-qPCR experiments. Data are means of at least 2 to 4 experiments for each antibody/amplicon combination (SE is provided when at least 3 experiments are averaged for a given antibody/amplicon). * indicates statistically significant difference between S0 and S1 at the HS2 site (*p* = 0.019 and 0.032 for H3K27me3 and H3K4me2, respectively). Changes in H3K27me3 and H3K4me2 over all HSs tested were significant at *p* = 0.011 and *p* = 0.0006, respectively (paired *t* test used for all significance tests). (G) Representative ChIP-qPCR experiment that included sorted S0, S1, and fetal brain. Data are mean ± SE of three replicates, expressed as a ratio of S1 to S0 (upper panel) and S0 to fetal brain (lower panel).

A long-standing hypothesis suggests that DNA replication may provide an opportunity for the restructuring of chromatin at tissue-specific gene loci [4],[5]. Given the requirement for DNA replication for the transition from S0 to S1, we asked whether chromatin change may be taking place at this time. The β-globin gene locus (Figure 5A) is a well-studied model of tissue-specific gene expression. The features that characterize the open chromatin conformation at the actively transcribed locus in erythroid cells have been established, but the time during development when the active chromatin conformation is acquired is not known. We therefore set out to examine whether the S0 to S1 transition might coincide with an alteration in the structure or function of chromatin at this locus.

The timing of replication of the β-globin locus is correlated with its chromatin state. In higher eukaryotes the timing of replication of genes correlates with their transcriptional activity [32]. Housekeeping genes replicate early in S-phase, whereas silent chromatin and heterochromatin replicate late. The β-globin locus replicates in mid to late S-phase in non-erythroid cells, and early in S-phase in erythroid cells [33]. We examined the timing of replication of the β-globin locus in S0 and S1 cells sorted from fresh fetal liver. Individual alleles were identified using fluorescence in situ hybridization (FISH) with a probe directed at the β-major gene. Cells in S-phase were identified by positive staining for BrdU incorporation. Nuclei from at least 100 S-phase cells from either S0 or S1 were examined in each of two experiments (Figure 5B). Using this approach, two single dots (“SS”) suggest that neither of the β-globin alleles had yet replicated. Nuclei in which both alleles have replicated contain a pattern of two double dots (“DD”). Replication of only one allele results in one single and one double dot (SD) [33]. We found that the number of cells with a DD pattern increased from only 15% in S0 to over 50% in S1 (Figure 5B), suggesting a switch in the timing of replication from late to early S-phase. In addition, an average of 36% of S0 cells, but only 21% of S1, had an SD pattern, consistent with a switch from late, asynchronous replication in S0 to early, synchronous replication in S1 [33].

### The S0 to S1 Transition Coincides with the Onset of DNase I Hypersensitivity at the β-Globin Locus Control Region (LCR)

A key indicator of open chromatin at the β-globin LCR is the presence of hypersensitivity (HS) sites (Figure 5A). We prepared nuclei from freshly sorted S0 or S1 cells and tested their sensitivity to DNase I digestion. Following digestion, we measured remaining DNA using quantitative PCR, with amplicons within HS2, HS3, and HS4 [34]. Results were expressed as a ratio to the DNase I resistant, non-expressing neural gene, Nfm. We found that S0 cells were relatively resistant to DNase I, while S1 cells were hypersensitive at all tested HS sites (Figure 5C). Therefore, the S0 to S1 transition coincides with the onset of DNase I hypersensitivity at the β-globin LCR.

We also examined E12.5 EpoR^−/−^ whole fetal livers, which do not contain S1 cells (Figure 1B). We found that EpoR^−/−^ fetal livers were resistant to DNase I, whereas whole fetal livers from wild-type or heterozygous littermates showed the expected hypersensitive sites (Figure 5D). We therefore concluded that DNase I hypersensitivity develops at the S0 to S1 transition, synchronously with the onset of EpoR dependence.

### S-Phase Progression Is Required for the Onset of DNase I Hypersensitivity at the β-Globin LCR

Since the transition from S0 to S1 coincides with, and requires, S-phase progression, we examined whether development of DNase I hypersensitivity at the β-globin LCR also requires S-phase progression. We incubated sorted S0 cells in Epo, in the presence or absence of aphidicolin, for 10 h. Over this period 25%–50% of S0 cells transition into S1, a process arrested by aphidicolin (Figures 1H, 2C). At the end of a 10-h incubation period, nuclei were prepared and digested with varying concentrations of DNase I. There was a clear increase in DNase I sensitivity in cells incubated in Epo alone, relative to cells incubated in Epo and aphidicolin (Figure 5E). Therefore, the development of DNase I hypersensitivity at the S0 to S1 transition is dependent on S-phase progression.

### Changes in Post-Translational Histone Tail Modifications Associated with the Transition from S0 to S1

The switch in timing of replication and in DNase I hypersensitivity at the S0 to S1 boundary suggested the β-globin LCR was undergoing structural changes. To investigate these, we used chromatin immunoprecipitation (ChIP) to determine specific histone tail modifications at the β-globin LCR in freshly sorted S0, S1, and in fetal brain. We used ChIP-qPCR for amplicons at the β-globin LCR HS sites, or at a control, neural gene, Nfm. Changes in histone modifications were expressed as a ratio, between S0 and either S1 or fetal brain (Figure 5F,G). Figure 5F summarizes data pooled from seven experiments with various immunoprecipitating antibodies as indicated. A comparison of S1 with S0 shows a 7-fold decrease in trimethylation of histone 3 lysine 27 (H3K27me3, *p* = 0.019, paired *t* test), a mark associated with silent chromatin, and a 2.5-fold increase in histone 3 lysine 4 dimethylation, a mark associated with active chromatin (H3K4me2, *p* = 0.032), at the HS2 site of the β-globin LCR. A similar trend for these two modifications was also found at other HS sites (*p* = 0.0006 and *p* = 0.011 for H3K4me2 and H3K27me3, respectively, pooling all HS sites). An increase in acetyl marks in histones H3 and H4 associated with active chromatin was also seen consistently across the HS sites tested, though it did not reach statistical significance. Of note, no significant changes in histone marks were found between S0 and S1 at the Nfm gene. Further, there was no significant change in total histone occupancy of the HS sites between S0 and S1, as determined by ChIP with antibodies directed against total H3 and H4 (Figure 5F).

We noted that H3K27me3, associated with silent chromatin, and H3K4me2, associated with active chromatin, were both enriched in S0 compared with fetal brain (Figure 5G, lower panel). These results were suggestive of bivalent chromatin at the β-globin LCR in S0, and loss of the repressive H3K27me3 mark with transition into S1 (Figure 5G, upper panel, 5F).

### The Transition from S0 to S1 Coincides with S-Phase-Dependent DNA Demethylation at the β-Globin LCR

**Figure 6:**
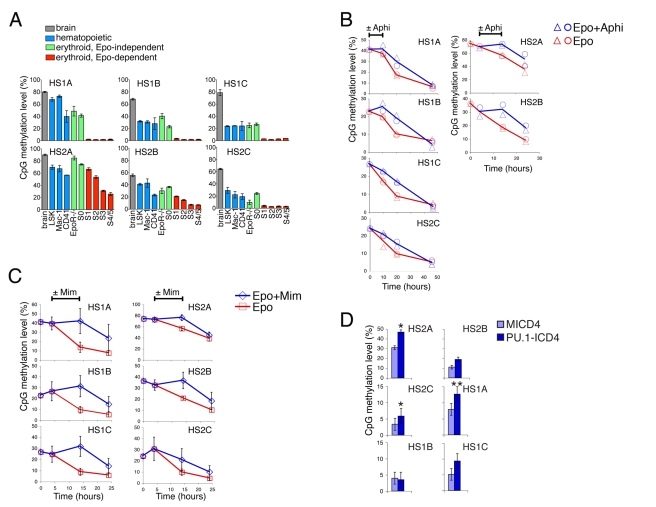
The transition from S0 to S1 is marked by the onset of S-phase dependent, DNA demethylation at HS1 and HS2. (A) Methylation levels at each of the 6 CpG dinucleotides in HS1 (HS1A, B, C) and HS2 (HS2A, B, C; Figure 5A), in each of the indicated cell populations. Hematopoietic cells were sorted flow-cytometrically from freshly isolated fetal liver. Brain  =  fetal brain; LSK  =  Lin^−^Sca1^+^Kit^+^; Mac-1 =  CD71^low^Ter119^−^Mac-1^+^; CD41 =  CD71^low^Ter119^−^CD41^+^; EpoR^−/−^  =  Lin^−^ cells from EpoR^−/−^ fetal liver. Methylation levels were assessed following PCR-amplification of bisufite-converted genomic DNA at each of the HS1 and HS2 loci followed by pyrosequencing. Each data point is the mean ± SE of 2 to 4 independent sorting and pyrosequencing experiments. (B) Arrest of S-phase progression by aphidicolin prevents DNA demethylation. Aphidicolin was added for 10 h at t = 0 (HS1A, B, C, and HS2C, left panel) or at t = 4 h (HS2A, B, right panel) to sorted S0 cells incubated in Epo for 48 (left panel) or 24 h (right panel). Control cells were incubated in Epo only. CpG methylation was measured at the indicated time points as described in (A). Data are from two independent experiments. (C) Arrest of S-phase entry by mimosine prevents DNA demethylation. Sorted S0 cells were incubated in Epo for 24 h, in the presence or absence of mimosine between t = 4 and t = 14 h. CpG methylation was measured as described in (A). Data are mean ± SE of three independent sorting and pyrosequencing experiments. (D) Preventing PU.1 downregulation prevents DNA demethylation of HS1 and HS2 at the S0 to S1 transition. S0 cells were transduced with retroviral vectors expressing either PU.1-ICD4 or MICD4 as described in Figure 4A–C. CpG methylation levels were measured as described in (A) following 24-h incubation in Epo. Data are mean ± SE of three independent experiments (cells were transduced with PU.1-ICD4 at >90% efficiency in one experiment, and in two additional experiments hCD4^+^ cells were sorted before the start of Epo incubation).

We examined DNA methylation of six CpG dinucleotides, three each at the HS1 and HS2 sites of the β-globin LCR (Figures 5A, 6A). Genomic DNA was prepared from sorted hematopoietic cell subsets from fresh fetal liver, including S0, S1, megakaryocytic CD41^+^, myeloid Mac-1^+^, and Lin^−^Sca1^+^Kit^+^ (LSK) cells, enriched for hematopoietic stem-cells. We also examined EpoR^−/−^ fetal livers depleted of cells expressing lineage markers, and fetal brain. DNA methylation at each of the six CpGs was obtained following bisufite conversion of genomic DNA, PCR amplification at HS1 and HS2, and pyrosequencing. In fetal brain methylation levels were high, at ≈60%–80%, for all six CpG dinucleotides. Methylation levels were lower in all hematopoietic cell subsets (Figure 6A). Methylation levels were largely similar in all hematopoietic, Epo-independent cell subsets examined: LSK, Mac-1^+^, CD41^+^, S0, and EpoR^−/−^ cells. The onset of Epo dependence in S1 was associated with a marked reduction in DNA methylation in all six CpG dinucleotides, with the level of methylation dropping to virtually undetectable levels in S1 for four of the six CpGs.

We found that DNA demethylation also took place in freshly sorted S0 cells allowed to differentiate in vitro (Figure 6B,C). Demethylation in vitro occurred earlier at the HS1A, B, C, and HS2C than at HS2A, B (Figure 6C, red lines), in agreement with results in vivo (Figure 6A). Demethylation in vitro was arrested at all CpGs if either aphidicolin or mimosine were added to the incubation medium, and resumed when these drugs were removed (Figure 6B,C). Therefore, DNA demethylation, initiated at the transition from S0 to S1, is dependent on S-phase progression. These results are suggestive of a passive demethylation process, due to loss of maintenance methylation at nascent DNA.

### PU.1 Downregulation Is Required for the Onset of DNA Demethylation at the Transition from S0 to S1

We examined HS1 and HS2 DNA methylation levels in S0 cells transduced with PU.1 (as in Figure 4A) and incubated in Epo for 24 h. DNA methylation was significantly higher at 3 of the 6 CpGs in S0 cells transduced with PU.1-ICD4, compared with control cells transduced with MICD4 (Figure 6D). Therefore, PU.1 expression, along with its inhibitory effect on erythroid differentiation, also impaired DNA demethylation, possibly due to its inhibitory effect on S-phase in these cells (Figure 4C).

## Discussion

**Figure 7:**
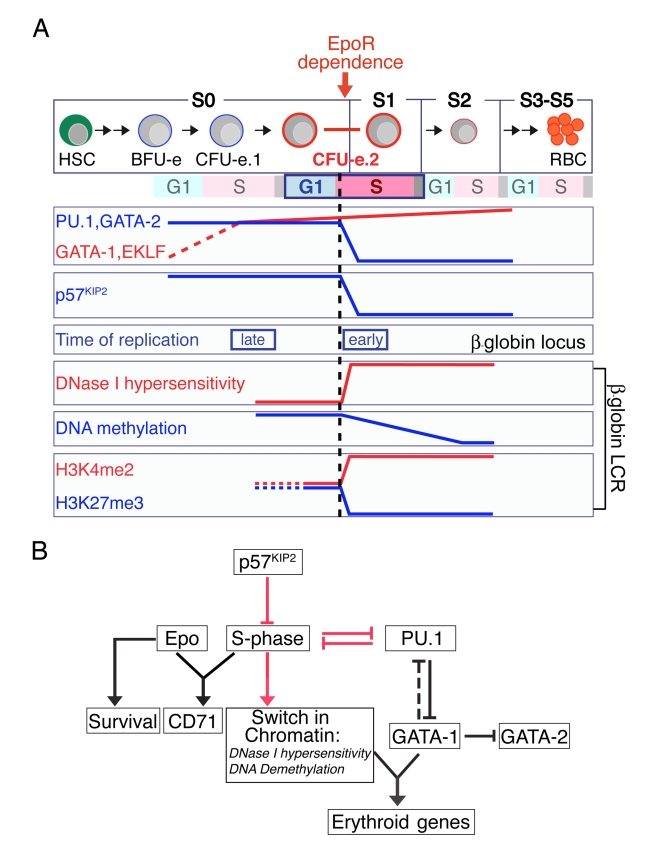
Regulatory events at the transition from S0 to S1. (A) *Multiple differentiation milestones coincide with early S-phase in the last CFU-e generation*. Upregulation of CD71 marks the transition from S0 to S1 in early S-phase of this cell cycle. It coincides with the onset of Epo dependence, downregulation of transcriptional suppressor PU.1 and CDKI p57^KIP2^, and reconfiguration of chromatin at the β-globin locus, including a switch in the timing of replication from late to early S-phase and, within the LCR, the formation of DNase I hypersensitivity sites, the onset of DNA demethylation, and a loss of repressive histone marks from bivalent chromatin. The dashed black line marks the time at which inhibition of S-phase arrests PU.1 downregulation, CD71 upregulation, and the switch in chromatin. Induction of erythroid transcriptional activators, including GATA-1, precedes this step. Erythroid specific genes including β-globin, Band3, and ALAS2 are induced subsequently. Upregulation of Ter119 occurs approximately with entry into the next cell cycle. (B) *Causal relationships at the transition from S0 to S1*. Red arrows mark novel causal relationships implicated in the transition from S0 to S1. Downregulation of p57^KIP2^ is required for S-phase progression during the last CFU-e generation. Mutual antagonism between PU.1 and S-phase results in PU.1 downregulation as cells progress into S-phase. Decreasing PU.1 allows for functional activation of GATA-1, which in turn represses GATA-2 and induces erythroid-specific genes such as β-globin. S-phase progression is also required for the formation of DNase I hypersensitive sites and for DNA demethylation bringing about a switch in chromatin conformation at the β-globin LCR and, together with Epo, for CD71 upregulation.

We have identified a committal step in erythropoiesis in which the cell cycle clock is precisely synchronized with and coordinates an erythroid differentiation switch. It takes place during S-phase of the last CFU-e generation, at the transition from S0 to S1, when S-phase progression is required for several distinct committal differentiation events, including the onset of Epo dependence, a switch in chromatin at the β-globin locus into an open conformation, and activation of GATA-1 function with consequent transcription of GATA-1 target genes (Figure 7A). The transition from S0 to S1 can be replicated in vitro, where sorted S0 cells develop into a differentiation state characteristic of S1 within 10 to 12 h.

S-phase progression at the S0 to S1 transition and the ensuing differentiation switch are dependent on the downregulation of p57^KIP2^ (Figures 3G, 7B), a novel finding since, to date, the principal known role of CDKIs in differentiating cells had been to mediate terminal differentiation secondary to cell cycle exit [1]–[3]. Unlike other CDKIs, p57^KIP2^ is required for the development of multiple tissues [35], suggesting that its novel role in erythropoiesis, triggering S-phase progression during a committal differentiation event, may be replicated in other systems. Of note, p57^KIP2^ is the only CDKI of the CIP/KIP family to be downregulated at the S0/S1 transition.

The synchronization of S-phase progression with several rapid and committal differentiation transitions suggests they are co-regulated. A key mediator of this co-regulation is PU.1, whose expression declines at the transition from S0 to S1. We have identified a novel cross-antagonism between S-phase progression and PU.1 expression. We show that S-phase arrest, caused by high levels of p57^KIP2^ or by cell cycle blocking drugs, prevents downregulation of PU.1 (Figure 3E,H); conversely, failure to downregulate PU.1 arrests S-phase progression (Figure 4B,C). Either maneuver blocks erythroid differentiation, including a block of chromatin reconfiguration at the β-globin locus and blocked expression of erythroid-specific genes (Figures 3B, 3D–H, S3G, S3I, 4B, 5E, 6C–D).

We propose that the mutual inhibition between PU.1 and S-phase progression at the S0 to S1 transition (Figure 7B) simultaneously controls the transition in both differentiation and cell cycle states. Its function is analogous to a synchromesh mechanism in automotive transmission, matching the speeds of two rotating gears before allowing them to lock together during a gear-shift. The mutual antagonism between S-phase progression and PU.1 expression ensures that PU.1 downregulation does not occur prior to the cell's entry into S-phase; conversely, S-phase entry cannot occur before conditions for PU.1 downregulation are in place. In this manner the cell cycle and differentiation programs can only proceed when precisely synchronized.

Once cells have transitioned from S0 into S1, S-phase progression is no longer required for expression of erythroid genes (Figure 3C, Figure S3E–F). Therefore, the synchromesh mechanism is specific to the transition from S0 to S1, when committal decisions bring about an irreversible terminal differentiation phase. Our findings reveal a key organizational feature in erythroid differentiation and have implications for differentiation of other lineages, where similar synchronization events may occur. Further, the synchromesh mechanism we describe may be a target in leukemogenesis, consistent with reports that high levels of PU.1 promote erythroleukemia [15]. Similarly, although no reports at present implicate p57^KIP2^ specifically in erythropoiesis, mutations in p57^KIP2^ are implicated in the familial Beckwith-Weidemann syndrome, which predisposes to pediatric tumors [22].

### The Cross-Antagonism between PU.1 and S-Phase Progression Is Lineage and Differentiation Stage-Specific

PU.1, whose physiological function in erythropoiesis had not been clear, plays a pivotal role at the S0 to S1 transition, through its cross-antagonism with S-phase progression. This cross-antagonism is lineage and differentiation stage-specific, since it presumably does not operate in myeloid and B-cell lineages where PU.1 is an essential transcriptional activator. Similarly, within the erythroid lineage, this mutual antagonism must be activated specifically in the last generation of CFU-e. Its premature activation at an earlier CFU-e cycle may be predicted to result in premature transition into S1 and consequently, in a reduced number of differentiated progeny. This prediction helps explain previous observations, where erythroid cells from PU.1-null embryos were found to differentiate prematurely and to have reduced self-renewal capacity [36]. These observations are consistent with the PU.1-null phenotype mimicking premature downregulation of PU.1. The mutual inhibition between PU.1 and S-phase may also explain findings in the T-cell lineage, where exogenous expression of PU.1 at the pro-T cell stage was found to block both thymocyte expansion and differentiation [37].

### The Cross-Antagonism between PU.1 and GATA-1

Previous work documented cross-antagonism between PU.1 and GATA-1, showing them to interfere with each other's transcriptional functions through a variety of mechanisms including direct physical binding [11]–[14]. We propose that the activation of erythroid terminal differentiation at the S0/S1 boundary is due to functional activation of GATA-1 (Figure 7B). Though present in S0 cells prior to the transition into S1 (Figure 3A), GATA-1 function is inhibited by PU.1. Downregulation of PU.1 at the S0 to S1 transition alleviates this inhibition, allowing GATA-1-mediated activation of erythroid gene induction. Among its known targets, GATA-1-mediated transcriptional repression of GATA-2 [29] would account for our observation that GATA-2 is downregulated at the S0 to S1 transition (Figure 3A). This scheme places the decrease in GATA-2 downstream of the cross-antagonism between PU.1 and GATA-1 (Figure 7B) and explains why exogenous high levels of GATA-2, unlike PU.1, do not block the transition from S0 to S1 (Figure 4).

Based largely on immortalized progenitor-like cells, the antagonism between GATA-1 and PU.1 was proposed to underlie a binary cell fate choice in cells expressing both GATA factors and PU.1. An increase in GATA-1 would result in PU.1 suppression and the erythro-megakaryocytic cell fates, whereas an increase in PU.1 would suppress GATA-1 and give rise to the myelo-lymphocytic lineages [11]–[14]. However, our data show that CFU-e cells, considered committed erythroid progenitors, express PU.1 at levels equivalent with those found in cells of the myeloid lineage (Figure S3A). Our results are consistent with previous reports of PU.1 expression in early erythroid progenitors, including the expression of a GFP reporter “knocked in” to the PU.1 gene locus in S0 (CD71^low^Ter119^negative^) fetal liver cells [36],[38].

The biochemical nature of commitment to the erythroid lineage is unknown at present. It is possible that CFU-e cells prior to PU.1 downregulation, which are also expressing GATA-1 and GATA-2, are in fact multipotential cells that may give rise to either myeloid or erythro-megakaryocytic lineages. In this case, the cross-antagonism between PU.1 and GATA-1 would simultaneously be responsible for a lineage choice, as well as facilitate activation of the erythroid gene expression program at the S0 to S1 transition should this choice be in favor of the erythroid lineage. Our ability to isolate CFU-e cells expressing high levels of PU.1 prior to their transition into S1 should facilitate further study of this issue.

### The Role of S-Phase

Why is S-phase progression coupled to the erythroid differentiation program at the S0 to S1 boundary? Linking developmental transitions to cell cycle phases may serve as a strategy for their correct developmental timing [39] and may ensure the correct number of differentiated progeny. Another possibility is that S-phase progression plays a direct role in the re-configuration of chromatin at erythroid-specific gene loci. DNA replication was proposed to provide an opportunity for structural changes in chromatin, since the passage of the replication fork transiently disrupts nucleosomes [4],[5]. Indeed, S-phase is essential for activation or silencing of some genes in yeast [40],[41] and metazoa [39],[42]–[45], though it is not known that this is due to a requirement in the reconfiguration of chromatin. However, S-phase is not required for activation of other developmental genes [46]–[50]. Further, in recent years the structure of chromatin was found to be much more dynamic outside S-phase than originally suspected [51]. It is therefore unclear whether there is an innate requirement for DNA replication in the reconfiguration of chromatin during activation of lineage-specific genes, or what specific aspects of chromatin restructuring might require S-phase.

Here we found that S-phase is required for DNA demethylation and for formation of DNase I hypersensitive sites. The requirement for DNA replication suggests that DNA demethylation is passive, due to a decrease in maintenance methylation of the nascent DNA strand [52]. This raises the possibility that formation of DNase I hypersensitive sites may require DNA replication because it might be contingent on DNA demethylation. Alternatively, DNase I hypersensitivity may require S-phase progression in order to lift a direct repressive effect of PU.1 on chromatin [13].

### The Role of EpoR

Our examination of EpoR^−/−^ fetal liver shows that the EpoR becomes essential for erythroid differentiation at the S0/S1 boundary. The principal function of EpoR at this time is its pro-survival signaling: EpoR^−/−^ erythroid progenitors undergo apoptosis but their cell cycle status is unaltered, suggesting that EpoR signaling is not required for S-phase progression (Figure S1E). These findings are consistent with the established role of EpoR as a survival factor that does not affect the erythroid cell cycle [53]. EpoR signaling is probably also dispensable for downregulation of PU.1 at the S0 to S1 transition, since both PU.1 and GATA-2 are low in EpoR^−/−^ cells (Figure 3A).

In spite of both S-phase progression and PU.1 downregulation being apparently unimpaired, EpoR^−/−^ cells fail to develop DNase I HS sites and fail to undergo DNA demethylation at the β-globin LCR (Figures 5D, 6A). It has been reported that exogenous expression of bcl-x_L_ facilitates Epo-independent differentiation of erythroblasts [54] arguing against a direct requirement for EpoR signaling in chromatin reconfiguration. Therefore, EpoR^−/−^ cells may be undergoing rapid apoptosis prior to the time when the chromatin change would have otherwise taken place.

Other than its survival function, EpoR is probably directly required for CD71 upregulation, via Stat5 [55],[56]. However, EpoR signaling results in CD71 upregulation only if S-phase is allowed to proceed (Figure 2).

Thus, while the onset of Epo dependence occurs synchronously with committal chromatin and transcriptional events in erythroid differentiation, there is apparently no direct requirement for EpoR signaling in these events, other than ensuring cell survival. The principal function of Epo in erythropoiesis is to determine the number of differentiated erythrocytes, via Epo concentration [57]. The S0 to S1 transition may have evolved as the time of onset of Epo dependence as it represents a biochemical commitment to erythroid differentiation, setting in motion chromatin and transcriptional transformations that lead to expression of erythroid-specific genes. This therefore represents the earliest time in erythroid differentiation when Epo may regulate cell number specifically within the erythroid lineage, with minimal lateral effects on other hematopoietic cells.

### An All-or-None Switch in Chromatin State at the β-Globin LCR

The β-globin LCR had long been studied as a model of chromatin at sites of lineage-specific genes. However, the time in erythroid differentiation when the locus switches from a “closed” to an “open” conformation had not been clearly defined. Further, it was not known whether activation of the locus develops in a step-wise fashion over several cell cycles and differentiation stages or whether it occurs rapidly in a single step.

Our findings show that, strikingly, the locus transitions to an active conformation rapidly, within S-phase of a single cell cycle. Further, several distinct functional and biochemical changes that characterize the active chromatin conformation appear to develop simultaneously. We found marked differences between S0 and S1 cells in DNA methylation and in DNase I hypersensitivity at the LCR. These transformations could be reproduced when purified S0 cells transitioned into S1 in vitro (Figures 5, 6). Both DNA demethylation and DNase I hypersensitivity required S-phase progression for their development. Further, we also found that development of histone-tail modifications characteristic of active chromatin, as well as the switch in the timing of replication of the locus from late to early S-phase, both coincide with the transition from S0 to S1 (Figure 5). Therefore, our findings support an “all or none” model for the state of chromatin, previously hypothesized based on the probabilistic nature of developing DNase I hypersensitivity in a range of mutated chicken β-globin enhancer constructs [58].

Previous work showed that while the highest levels of DNase I accessibility at the β-globin LCR are attained in mature erythroid progenitors, the β-globin LCR is already poised for expression in earlier multipotential progenitors, contributing to low-level β-globin transcription (“priming”) [59],[60]. The β-globin LCR was found to already contain DNase I hypersensitive sites in cell lines resembling early hematopoietic progenitors [61]. Here we find that the β-globin LCR appears poised for change prior to the transition from S0 to S1. Thus, LSK and S0 cells have similar DNA methylation levels that are substantially lower than in fetal brain, suggesting chromatin already primed for expression at the LSK stage (Figure 6A). Histone tail modifications in the LCR similarly suggest that chromatin in S0 is poised for change, as it is enriched with both H3K4me2, a mark associated with active chromatin, and with H3K27me3, a mark found in silent chromatin (Figure 5G). The LCR is therefore marked as a bivalent domain, which may denote chromatin that is silent but primed for activation [62],[63]. Regardless of the precise state of chromatin readiness in earlier hematopoietic progenitors, however, our results show a clear switch in chromatin conformation at the S0 to S1 transition.

The clear switch we identified at the β-globin LCR occurs in synchrony with other switch-like transformations at the transition from S0 to S1, including the onset of Epo dependence and activation of GATA-1 function. Our ability to identify this transition with precision in vivo and manipulate it genetically in vitro should facilitate further study of the pivotal link between the cell cycle clock and the committal chromatin decisions that bring about the erythroid phenotype.

## Materials and Methods

### Flow Cytometry

Fetal livers (E12.5–E14) were mechanically dissociated and immunostained as described [64]. Immunofluorescence was measured on an LSRII (BD Biosciences, CA) and data analyzed using FloJo (Tree Star, CA). Cells were sorted on a FACSAria, FACSVantage (BD Biosciences), or MoFlo (Beckman Coulter) cell sorters. In a small number of experiments StemSep columns (StemCell Technologies) were used.

### In Vitro Culture

Freshly harvested fetal liver cells were sorted and cultured in medium containing 20% fetal calf serum and 2 U/ml Epo (Amgen) for up to 48 h.

### Cell Cycle Analysis

BrdU (100 µl of 10 mg/ml) was injected intra-peritonealy to pregnant mice and embryos were harvested 30–50 min later. In vitro, cells were pulsed with BrdU for 30 min. BrdU incorporation was detected using BrdU flow kit (BD Biosciences). Cell tracking with CFSE (carboxyfluorescein diacetate succinimidyl ester) was performed on sorted S0, incubated with 2.5 µM CFSE (Invitrogen) for 10 min at 37°C.

### Retroviral Transduction

Retroviral transduction was by spin infection of sorted S0 cells at 2,000 rpm, 37°C on fibronectin coated dishes in 5 µg/ml polybrene (Sigma). Transduced cells were incubated overnight in the presence of 100 ng/ml SCF and 10 ng/ml IL3 (Peprotech, Rocky Hill, NJ) and were then transferred to Epo-containing medium for the indicated times.

### Quantitative RT-PCR

Quantitative RT-PCR was performed as described [64].

### DNase I Hypersensitivity Assays

DNase I hypersensitivity assays were performed as described [34] with modifications to amplicons (see Supplemental Methods).

### ChIP-qPCR

ChIP-qPCR was performed on 10^6^ cells/sample of sorted S0, sorted S1, or fetal brain. Cells were cross-linked in 1% formaldehyde, sonicated, and incubated overnight with a range of antibodies (see Supplemental Data), followed by 3–4 h of incubation with Protein G-magnetic beads (Invitrogen). Cross-links were reversed and purified DNA measured by qPCR using the same amplicons as in the DNase I hypersensitivity assay.

### DNA Methylation

Genomic DNA or cells were treated with sodium bisulfite (Zymo Research, Orange, CA). Bisulfite-converted DNA was amplified by PCR and methylation levels measured using pyrosequencing at EpigenDx (Worcester, MA).

See Text S1 for additional methods, primer sequences, and antibodies.

## Supporting Information

Figure S1 **Supplemental data to** **Figure 1** **.** (A) Ter119^+^ cells in EpoR^−/−^ fetal livers are nucleated erythrocytes of the yolk-sac (primitive) lineage. Cytospin preparations of sorted Ter119^+^ cells from fetal livers of EpoR^−/−^ (E12.5) and wild-type littermate. Yolk-sac erythrocytes are fully hemoglobinized, large nucleated cells (arrow; see brown coloration of hemoglobinized cells stained with diaminobenzidine). Basophilic (blue cytoplasm) erythroblast precursors of the definitive lineage form the majority of Ter119^+^ cells in the wild type fetal liver but are absent from EpoR^−/−^ fetal liver. Scale bar  = 20 µ. (B) Distribution of cells expressing non-erythroid lineage markers within fetal liver. The fraction of cells expressing each indicated marker is shown for embryonic ages E12.5 to E14.5 in wild-type embryos, and for EpoR^−/−^ embryos on E12.5. The same data are represented for each lineage marker either as a fraction of whole fetal liver (left panel) or as a fraction of S0 (right panel). (C) The fraction of S0 cells expressing EpoR mRNA assessed by single cell RT-PCR. Single cell RT-PCR was carried out on 324 individual S0 cells. An mRNA signal (either EpoR or β-actin or both) was obtained for 159 cells (49%). 68% of cells with a positive mRNA signal were positive for the EpoR mRNA. Single S0 cells were sorted by flow-cytometry into single wells of a 96-well plate. Following reverse transcription, two rounds of PCR amplification were used to detect EpoR and β-actin expression in individual cells. Shown are representative examples of RT-PCR for EpoR (top gel) and β-actin (lower gel) for four individual cells (lanes 1 to 4). The cells in lanes 1 and 4 expressed both EpoR and β-actin. Neither EpoR nor β-actin signals were obtained for the cell in lane 3. The cell in lane 2 expressed only β-actin. Control lanes are RT-PCR on spleen cells (positive control, “c1”), thigh muscle (negative control, “c2”), and no template (negative control, “c3”). (D) BrdU incorporation rate (measured as median fluorescence intensity, MFI) in wild-type S1 is higher than in other fetal liver subsets, and higher than in EpoR^−/−^ fetal liver (latter computed for the few cells within the S1 gate). Difference between wild-type S0 and S1 is significant at *p*<0.0001 (paired *t* test). Data are mean ± SE of 7 independent experiments. (E) Left panels: EpoR does not regulate cell cycle status of erythroid progenitors. Representative CD71/Ter119 profiles (upper panels) and BrdU/7AAD (lower panel) for the S0 subset in EpoR^−/−^ fetal liver and in wild-type littermates. The fractions (%) of S-phase cells (lower panels) is indicated. Right panel: EpoR regulates survival of erythroid progenitors. “LIVE/DEAD” profiles of the same EpoR^−/−^ and wild-type littermate embryos. The LIVE/DEAD dye (Molecular Probes) stains apoptotic cells with impaired membrane permeability prior to fixation and permeabilization assays. The fractions (%) of apoptotic cells are indicated. (F) qRT-PCR analysis of mRNA expression for p27^KIP1^, p21^CIP1^, and p57^KIP2^ in sorted fetal liver subsets S0 to S3 cells. Data were normalized to the β-actin mRNA in each sample and expressed as a ratio to the S0 subset. Data are mean ± SD of three independent experiments.
(0.90 MB TIF)

Figure S2 **Supplemental data to** **Figure 2** **.** (A) S1 cells are sensitive to hydroxyurea (HU). S0 cells were sorted by flow-cytometry (t = 0) and incubated in the presence or absence of Epo, and in the presence or absence of HU (5 mM), as indicated. Samples incubated in Epo alone upregulated CD71 and Ter119 sequentially (see t = 15 h, t = 24 h). Upregulation of CD71, but not Ter119, is Epo dependent (see “no Epo” sample in which Epo was added at t = 15 h). Cells incubated in Epo and HU did not upregulate CD71 or Ter119, consistent with the transition into S1 (CD71 upregulation) being S-phase dependent. Cells incubated in Epo alone for 15 h had transitioned into S1 and began to transition into S2. If HU was added at this point (t = 15 h), nearly all S1 cells are lost (due to HU toxicity, unpublished data), but many cells in S0 and S2 persist. This suggests that essentially all S1 cells are in S-phase. (B) Preventing CD71 upregulation at the S0/S1 boundary does not interfere with the erythroid cell cycle. Sorted S0 cells were transduced with retroviral vectors containing an “IRES-GFP” reporter, expressing short hairpin RNA targeting CD71 (CD71shRNA) or “empty vector” control (LMPv). Cells were then cultured for 24 h in the presence of Epo. Shown are the CD71/Ter119 profiles (top panels) and corresponding BrdU/7AAD cell cycle profiles of cells at t = 24 h. Only retrovirally infected cells are shown, identified by the expression of GFP. The fraction (%) of cells in each gate is indicated. Representative of three experiments. (C) Cytospin preparations of cells in the experiment described in (B) at t = 24 h. Control cells, but not cells expressing shRNA to CD71, have started to express hemoglobin in their cytoplasm (brownish color, see arrow). Stained with Giemsa-diaminobenzidine; scale bar is 20 µ.
(1.38 MB TIF)

Figure S3 **Supplemental data to** **Figure 3** **.** (A) Quantitative RT-PCR analysis of PU.1, Gata-2, and Gata-1 mRNAs in Mac-1^+^, S0, or S1 cells sorted from fetal liver. mRNAs were normalized to the β-actin mRNA in each sample. Data are mean ± SD of 2 independent experiments. (B) Total RNA per cell in sorted fetal liver subsets. Total isolated RNA for each subset was measured using spectrophotometric optical density and divided by the number of sorted cells. Mean ± SE of five independent sort experiments. (C) Flow-cytometry histograms of PU.1 protein levels in sorted fetal liver subsets S0 to S3. Fresh fetal liver cells were fixed, permeabilized, and stained for CD71, Ter119, and PU.1. Bar graph indicates the PU.1 median fluorescence intensity of each subset. Representative of 2 independent experiments. (D) EpoR mRNA increases at the S0 to S1 transition. Results normalized to β-actin and expressed as a ratio to S0. (E,F) Experimental design for (E) and (F): sorted S1 cells (t = 0 h) were incubated in Epo and in the presence or absence of aphidicolin, for 10 h. Cells were then washed free of aphidicolin and Epo incubation continued for a further 10 h. (E) Arrest of S-phase progression in S1 does not affect the mRNA expression of erythroid-specific genes, β-globin, Alas2, and Band3. mRNA was measured by qRT-PCR, normalized to β-actin, and expressed as relative to mRNA at t = 0. Data are mean ± s.e.m. from 3 independent experiments. (F) Arrest of S-phase progression in S1 does not affect expression of Ter119. CD71/Ter119 profiles showing that Ter119 was upregulated between t = 0 and t = 10 h regardless of the presence of aphidicolin. The fraction (%) of cells in S1 (left gate) or S2 (right gate) is indicated. Note that the larger cell size resulting from aphidicolin-mediated block of DNA replication (Figure 3F, main manuscript) is likely responsible for the higher Ter119 signal in the aphidicolin treated cells (cell surface Ter119 would be expected to increase in proportion to the square of the cell's radius). Ten hours following the release of the block, there is no significant difference in Ter119 expression between treated and untreated cell samples (t = 20 h). (G) Effect of mimosine-mediated S-phase arrest on downregulation of PU.1 and Gata-2 during the S0 to S1 transition. Experiment and mRNA measurement were as in Figure 3D, main manuscript, with the exception that mimosine was used in place of aphidicolin. Data are mean ± SE of three independent experiments. (H) Effect of aphidicolin-mediated S-phase arrest on Lmo2 and Nfe2 mRNAs during the S0 to S1 transition. Experiment and mRNA measurements as in Figure 3B, main manuscript; mRNA levels are expressed as a ratio to mRNA at t = 10 h in the “Epo only” control. Data are from two independent experiments. (I) Erythroblast morphology of S0 cells transduced with p57T329A-ICD4 or with control vector MICD4 at t = 32 h of incubation in Epo. Experiment as described in Figure 3G, main manuscript. Cytospins were stained with Giemsa-diaminobenzidine; scale bar = 20 µ.
(0.66 MB TIF)

Figure S4 **Supplemental data to** **Figure 4** **.** (A–B) PU.1 protein levels in cells transduced with PU.1-IRES-hCD4 is proportional to the level of the hCD4 reporter in the same cells. Please see also summary of these data in Figure 4D. (A) Expression profiles of the hCD4 reporter in cells transduced with either PU.1-ICD4, or MICD4 control vector, or uninfected control cells, following 24 h of culture in Epo. Vertical, narrow gates each containing cells of relatively uniform hCD4 expression, numbered 1 to 4, are shown and are used in the analysis in sections (B) and in Figure 4D. (B) Flow-cytometry histograms of cells transduced with MICD4 control vector (top panels) or with PU.1-ICD4 (lower panels) for each individual hCD4 gate (numbered 1 to 4), showing the percent of PU.1 positive cells. An overlay of the same flow-cytometry histograms is shown on the right. (C) Expression of GATA-1-IRES-hCD4 (GATA-1-ICD4), GATA-2-ICD4, and MICD4 at 24 h of Epo culture. The hCD4^+^ cell gate is indicated in black. Associated with Figure 4E in the main text. (D) qRT-PCR analysis of Gata-1, Gata-2, and PU.1 mRNAs in S0 cells transduced with MICD4 control vector or gene-ICD4 at 24 h of Epo culture, as compared with endogenous levels in freshly sorted S0 and S1 subsets. mRNA was normalized to the β-actin mRNA and expressed as a ratio to the S0 subset. Data are mean ± SD of 3 independent experiments (the transduction efficiency was >90% for PU.1-ICD4 and >50% for Gata-1-ICD4 and Gata-2-ICD4). (E) Preventing downregulation of PU.1 does not halt downregulation of p57^KIP2^ mRNA. Quantitative RT-PCR analysis of p57^KIP2^ mRNA in S0 cells retrovirally transduced with PU.1-ICD4 or with control vector MICD4 as described in Figure 4A–C, and incubated for 24 h in Epo. mRNA measurements from two independent experiments. In the first experiment, hCD4-positive cells were sorted by flow-cytometry at t = 0 (first experiment). In the second experiment transduction efficiency exceeded 90%, as judged by hCD4 expression. mRNAs were also measured in freshly sorted S0 and S1 subsets.
(0.55 MB TIF)

Text S1 **Supporting materials and methods.** Additional methods, primer sequences, and details of antibodies used.
(0.14 MB DOC)

## Abbreviations

BrdU: bromodeoxyuridine
CDKI: cyclin-dependent kinase inhibitor
CFSE: carboxyfluorescein diacetate succinimidyl ester
CFU-e: colony forming unit-erythroid
ChIP: chromatin immunoprecipitation
DD: double dots
FISH: fluorescence in situ hybridization
HS: hypersensitivity
IL-3: interleukin 3
IRES: internal ribosomal entry site
LCR: locus control region
SCF: stem-cell factor
SD: one single and one double dot
SS: two single dots
