# Supplementary figures and images for: A Key Commitment Step in Erythropoiesis Is Synchronized with the Cell Cycle Clock through Mutual Inhibition between PU.1 and S-Phase Progression
Source: PLoS Biol. 2010 Sep 21;8(9):e1000484. doi: 10.1371/journal.pbio.1000484 (PMC2943437; doi:10.1371/journal.pbio.1000484)

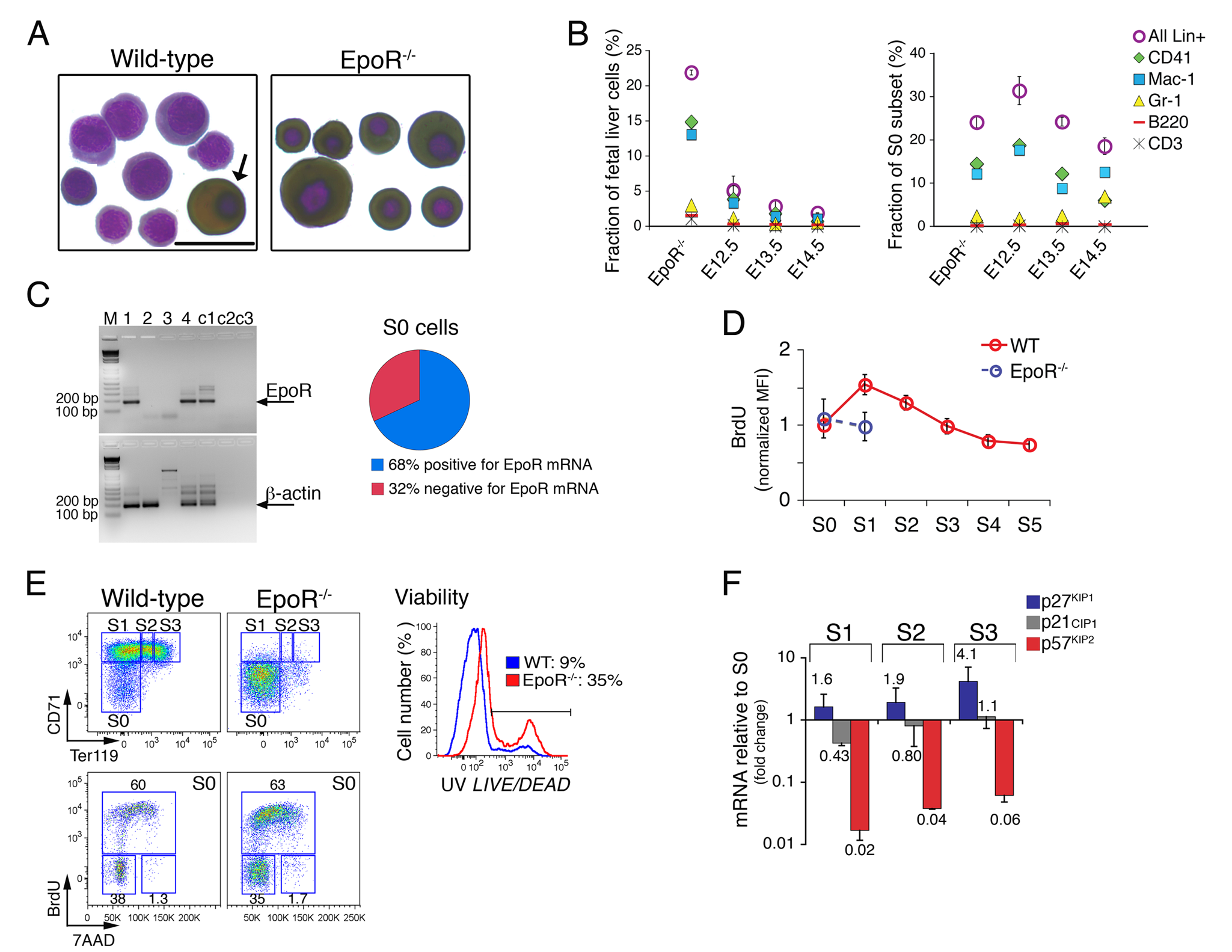

Supplement: Figure S1 — Supplemental data to Figure 1 . (A) Ter119+ cells in EpoR−/− fetal livers are nucleated erythrocytes of the yolk-sac (primitive) lineage. Cytospin preparations of sorted Ter119+ cells from fetal livers of EpoR−/− (E12.5) and wild-type littermate. Yolk-sac erythrocytes are fully hemoglobinized, large nucleated cells (arrow; see brown coloration of hemoglobinized cells stained with diaminobenzidine). Basophilic (blue cytoplasm) erythroblast precursors of the definitive lineage form the majority of Ter119+ cells in the wild type fetal liver but are absent from EpoR−/− fetal liver. Scale bar = 20 µ. (B) Distribution of cells expressing non-erythroid lineage markers within fetal liver. The fraction of cells expressing each indicated marker is shown for embryonic ages E12.5 to E14.5 in wild-type embryos, and for EpoR−/− embryos on E12.5. The same data are represented for each lineage marker either as a fraction of whole fetal liver (left panel) or as a fraction of S0 (right panel). (C) The fraction of S0 cells expressing EpoR mRNA assessed by single cell RT-PCR. Single cell RT-PCR was carried out on 324 individual S0 cells. An mRNA signal (either EpoR or β-actin or both) was obtained for 159 cells (49%). 68% of cells with a positive mRNA signal were positive for the EpoR mRNA. Single S0 cells were sorted by flow-cytometry into single wells of a 96-well plate. Following reverse transcription, two rounds of PCR amplification were used to detect EpoR and β-actin expression in individual cells. Shown are representative examples of RT-PCR for EpoR (top gel) and β-actin (lower gel) for four individual cells (lanes 1 to 4). The cells in lanes 1 and 4 expressed both EpoR and β-actin. Neither EpoR nor β-actin signals were obtained for the cell in lane 3. The cell in lane 2 expressed only β-actin. Control lanes are RT-PCR on spleen cells (positive control, “c1”), thigh muscle (negative control, “c2”), and no template (negative control, “c3”). (D) BrdU incorporation rate (measured a [file pbio.1000484.s001.tif]

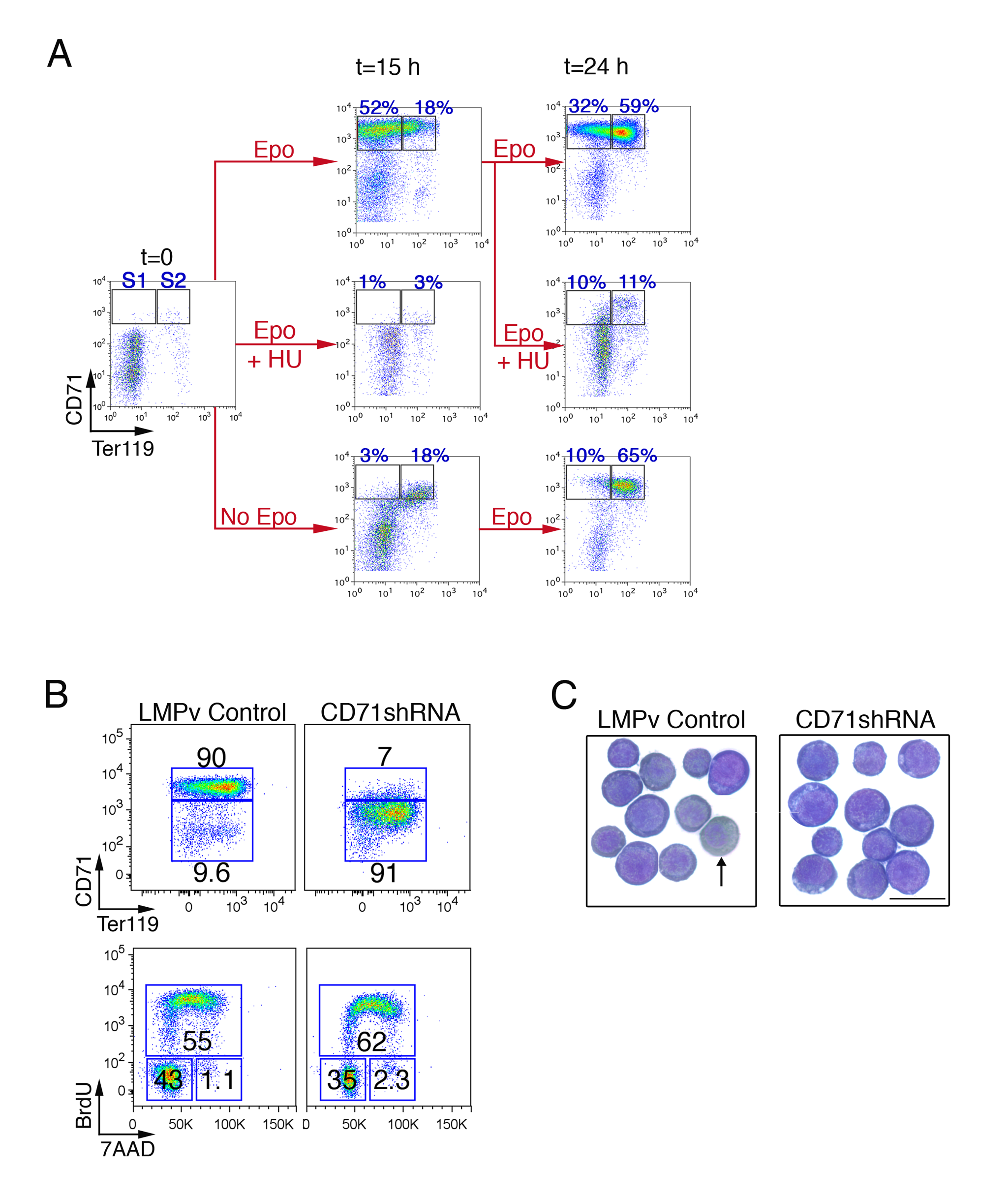

Supplement: Figure S2 — Supplemental data to Figure 2 . (A) S1 cells are sensitive to hydroxyurea (HU). S0 cells were sorted by flow-cytometry (t = 0) and incubated in the presence or absence of Epo, and in the presence or absence of HU (5 mM), as indicated. Samples incubated in Epo alone upregulated CD71 and Ter119 sequentially (see t = 15 h, t = 24 h). Upregulation of CD71, but not Ter119, is Epo dependent (see “no Epo” sample in which Epo was added at t = 15 h). Cells incubated in Epo and HU did not upregulate CD71 or Ter119, consistent with the transition into S1 (CD71 upregulation) being S-phase dependent. Cells incubated in Epo alone for 15 h had transitioned into S1 and began to transition into S2. If HU was added at this point (t = 15 h), nearly all S1 cells are lost (due to HU toxicity, unpublished data), but many cells in S0 and S2 persist. This suggests that essentially all S1 cells are in S-phase. (B) Preventing CD71 upregulation at the S0/S1 boundary does not interfere with the erythroid cell cycle. Sorted S0 cells were transduced with retroviral vectors containing an “IRES-GFP” reporter, expressing short hairpin RNA targeting CD71 (CD71shRNA) or “empty vector” control (LMPv). Cells were then cultured for 24 h in the presence of Epo. Shown are the CD71/Ter119 profiles (top panels) and corresponding BrdU/7AAD cell cycle profiles of cells at t = 24 h. Only retrovirally infected cells are shown, identified by the expression of GFP. The fraction (%) of cells in each gate is indicated. Representative of three experiments. (C) Cytospin preparations of cells in the experiment described in (B) at t = 24 h. Control cells, but not cells expressing shRNA to CD71, have started to express hemoglobin in their cytoplasm (brownish color, see arrow). Stained with Giemsa-diaminobenzidine; scale bar is 20 µ. (1.38 MB TIF) [file pbio.1000484.s002.tif]

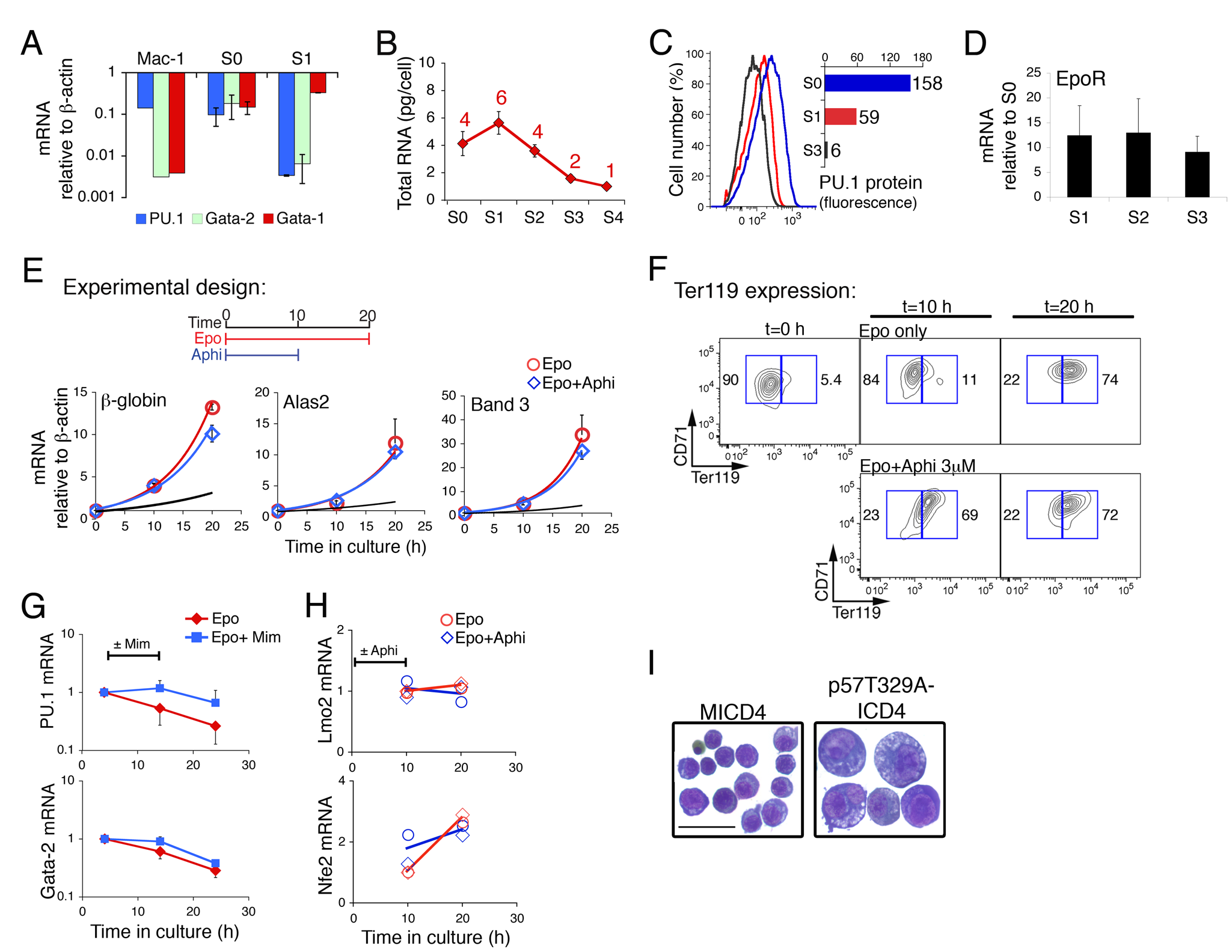

Supplement: Figure S3 — Supplemental data to Figure 3 . (A) Quantitative RT-PCR analysis of PU.1, Gata-2, and Gata-1 mRNAs in Mac-1+, S0, or S1 cells sorted from fetal liver. mRNAs were normalized to the β-actin mRNA in each sample. Data are mean ± SD of 2 independent experiments. (B) Total RNA per cell in sorted fetal liver subsets. Total isolated RNA for each subset was measured using spectrophotometric optical density and divided by the number of sorted cells. Mean ± SE of five independent sort experiments. (C) Flow-cytometry histograms of PU.1 protein levels in sorted fetal liver subsets S0 to S3. Fresh fetal liver cells were fixed, permeabilized, and stained for CD71, Ter119, and PU.1. Bar graph indicates the PU.1 median fluorescence intensity of each subset. Representative of 2 independent experiments. (D) EpoR mRNA increases at the S0 to S1 transition. Results normalized to β-actin and expressed as a ratio to S0. (E,F) Experimental design for (E) and (F): sorted S1 cells (t = 0 h) were incubated in Epo and in the presence or absence of aphidicolin, for 10 h. Cells were then washed free of aphidicolin and Epo incubation continued for a further 10 h. (E) Arrest of S-phase progression in S1 does not affect the mRNA expression of erythroid-specific genes, β-globin, Alas2, and Band3. mRNA was measured by qRT-PCR, normalized to β-actin, and expressed as relative to mRNA at t = 0. Data are mean ± s.e.m. from 3 independent experiments. (F) Arrest of S-phase progression in S1 does not affect expression of Ter119. CD71/Ter119 profiles showing that Ter119 was upregulated between t = 0 and t = 10 h regardless of the presence of aphidicolin. The fraction (%) of cells in S1 (left gate) or S2 (right gate) is indicated. Note that the larger cell size resulting from aphidicolin-mediated block of DNA replication (Figure 3F, main manuscript) is likely responsible for the higher Ter119 signal in the aphidicolin treated cells (cell surface Ter119 would be expected to increase in proportion to the square [file pbio.1000484.s003.tif]

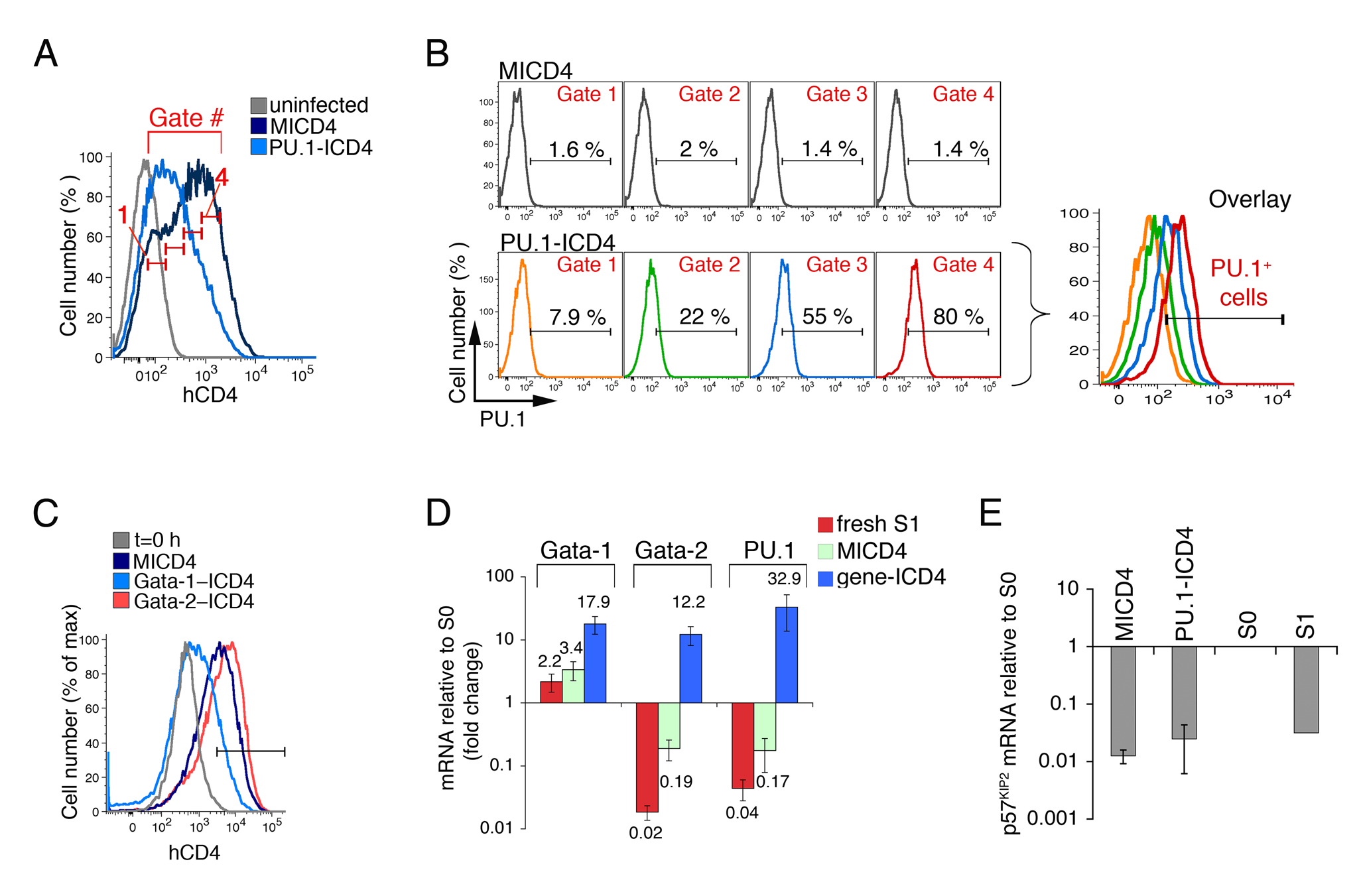

Supplement: Figure S4 — Supplemental data to Figure 4 . (A–B) PU.1 protein levels in cells transduced with PU.1-IRES-hCD4 is proportional to the level of the hCD4 reporter in the same cells. Please see also summary of these data in Figure 4D. (A) Expression profiles of the hCD4 reporter in cells transduced with either PU.1-ICD4, or MICD4 control vector, or uninfected control cells, following 24 h of culture in Epo. Vertical, narrow gates each containing cells of relatively uniform hCD4 expression, numbered 1 to 4, are shown and are used in the analysis in sections (B) and in Figure 4D. (B) Flow-cytometry histograms of cells transduced with MICD4 control vector (top panels) or with PU.1-ICD4 (lower panels) for each individual hCD4 gate (numbered 1 to 4), showing the percent of PU.1 positive cells. An overlay of the same flow-cytometry histograms is shown on the right. (C) Expression of GATA-1-IRES-hCD4 (GATA-1-ICD4), GATA-2-ICD4, and MICD4 at 24 h of Epo culture. The hCD4+ cell gate is indicated in black. Associated with Figure 4E in the main text. (D) qRT-PCR analysis of Gata-1, Gata-2, and PU.1 mRNAs in S0 cells transduced with MICD4 control vector or gene-ICD4 at 24 h of Epo culture, as compared with endogenous levels in freshly sorted S0 and S1 subsets. mRNA was normalized to the β-actin mRNA and expressed as a ratio to the S0 subset. Data are mean ± SD of 3 independent experiments (the transduction efficiency was >90% for PU.1-ICD4 and >50% for Gata-1-ICD4 and Gata-2-ICD4). (E) Preventing downregulation of PU.1 does not halt downregulation of p57KIP2 mRNA. Quantitative RT-PCR analysis of p57KIP2 mRNA in S0 cells retrovirally transduced with PU.1-ICD4 or with control vector MICD4 as described in Figure 4A–C, and incubated for 24 h in Epo. mRNA measurements from two independent experiments. In the first experiment, hCD4-positive cells were sorted by flow-cytometry at t = 0 (first experiment). In the second experiment transduction efficiency exceeded 90%, as judged by hCD4 expression. mRN [file pbio.1000484.s004.tif]
