# Supplementary material for: A Key Commitment Step in Erythropoiesis Is Synchronized with the Cell Cycle Clock through Mutual Inhibition between PU.1 and S-Phase Progression
Source: PLoS Biol. 2010 Sep 21;8(9):e1000484. doi: 10.1371/journal.pbio.1000484 (PMC2943437; doi:10.1371/journal.pbio.1000484)
Supplement: Text S1 — Supporting materials and methods. Additional methods, primer sequences, and details of antibodies used. (0.14 MB DOC) [file pbio.1000484.s005.doc]

### Supporting materials and methods

### Flow-cytometry of erythroid progenitors

Fetal liver cells were simultaneously stained for CD71, Ter119 and a Lineage-cocktail with anti-CD3, Gr-1, B220, CD41 and Mac-1 antibodies (BD Biosciences). Background fluorescence in each channel (‘fluorescence minus one’) was determined with isotype-matched antibodies substituting for each specific antibody, one channel at a time. Cells were sorted on a FACSAria, FACSVantage (BD Biosciences) or MoFlo (Beckman Coulter) cell sorters. Dead cells were excluded using 7-aminoactynomycin (7-AAD, Viaprobe, BD Biosciences) or DAPI (Roche). Alternatively, fetal liver cells were labeled with biotin-conjugated antibodies against CD71, Ter119, Gr-1, Mac-1 and CD41, and S0 cells were purified through a StemSep magnetic column as per manufacturer’s instructions (StemCell Technologies, Vancouver, BC, Canada).

For detection of PU.1 protein, fetal liver cells freshly sorted, or after retroviral infection were fixed and permeabilized using the BD Cytofix/Cytoperm kit (BD Pharmigen), stained for PU.1 with anti-PU.1 Alexa Fluor® 647 (#2240, Cell Signaling) and analysed by flow cytometry. FMO control with Rabbit (DA1E) mAb IgG Isotype Control Alexa Fluor® 647 (#2985, Cell Signaling) was used.

The following antibodies were used:

Rat Anti-Mouse Gr1-biotin monoclonal RB6-8C5, BD Pharmingen

Rat Anti-Mouse CD71-biotin monoclonal C2F2, BD Pharmingen

Rat Anti-Mouse Ter119-biotin monoclonal Ter119, BD Pharmingen

PE Anti-human CD4 monoclonal RPA-T4, BD Pharmingen

FITC Rat Anti-Mouse CD41 MWReg30, BD Pharmingen

FITC Rat Anti-Mouse CD45R/B220 RA3-6B2, BD Pharmingen

FITC Hamster Anti-Mouse CD3e 145-2C11, BD Pharmingen

FITC Rat Anti-Mouse CD411b/Mac-1 M1/70, BD Pharmingen

FITC Rat Anti-Mouse Ly-6G and Ly-6C (Gr-1) RB6-8C5, BD Pharmingen

Rat Anti-Mouse CD41-biotin monoclonal MWReg30, GenWay Biotech,Inc

PU.1 (9G7) Rabbit mAb Alexa Fluor® 647 (#2240, Cell Signaling)

**Retroviral vectors**

cDNA for PU.1 (provided by Tennen D., Beth Israel Deaconess Medical Center), GATA-1 and GATA-2 (Open Biosystems) were subcloned into MSCV-IRES-hCD4 retroviral vector.

p57T329A has a 1 bp mutation at position T329A (based on the human mutant p57 T310A, Kamura et al., 2003) which was created by PCR with the following primers: wild-type forward: 5’-ATGTCAGAATTCACAGCGATGGAACG-3’ and mutant reverse: 5’-ATGTCAGCGGCCGCTCATCTCAGACGTTTGCGCGGGGCCTGCTCC-3’. The mutation was verified by sequencing. CD71 shRNA (source ID V2MM_13414) was obtained through the UMass Medical School shRNA core facility and subcloned into the LMP-IRES-GFP retroviral vector (Open Biosystems). High-titer viral supernatants were prepared by co-transfecting the desired plasmid and the pCL-Eco packaging vector into Phoenix cells.

### Quantitative RT-PCR Assay

RNA was prepared using the AllPrep DNA/ RNA Micro Kit (Qiagen), and measured with RiboGreen RNA reagent kit (Thermo Scientific) on the 3300 NanoDrop Fluorospectrometer. Reverse-transcription was done using the SuperScript II/III (Invitrogen) with random hexamer primers. The ABI 7300 sequence detection system, TaqMan reagents and TagMan MGB probes (Applied Biosystems, San Diego, CA) were used following the manufacturer’s instruction. The ΔCt method was used to estimate mRNA using β-actin as a control.

The following TagMan MGB probes were used:

β-actin (actin, beta) Mm02619580_g1

BAND 3 (solute carrier family 4 anion exchanger) Mm01245920_g1

ALAS2 (aminolevulinic acid synthase 2, erythroid) Mm01260713_m1

PU.1 (SFFV proviral integration1) Mm00488140_m1

GATA-2 (GATA binding protein 2) Mm00492300_m1

GATA-1 (GATA binding protein 1) Mm01352636_m1

NF-E2 (nuclear factor, erythroid derived 2)  Mm00801891_m1

Tal1 (T-cell acute lymphocytic leukemia 1) Mm00441665_m1

LMO2 (LIM domain only 2) Mm00493153_m1

EKLF1 (Kruppel-like factor 1, erythroid) Mm00516096_m1

P57 (cyclin-dependent kinase inhibitor 1C) Mm00438170_m1

P21 (cyclin-dependent kinase inhibitor 1A) Mm00432448_m1

P27 (cyclin-dependent kinase inhibitor 1B) Mm00438168_m1

EpoR (erythropoietin receptor) Mm01175894_m1

### DNase I Hypersensitivity Assays

Nuclei were prepared by incubating cells for 5 minutes in hypotonic buffer (=’RSB’, 10 mM Tris pH 7.5, 10 mM NaCl, 3 mM MgCl2), then adding 0.2% NP40 and briefly vortexing. Nuclei were visually inspected, washed in DNase I digestion buffer (=’DDB’, 15 mM Tris pH 7.5, 15 mM NaCl, 60 mM KCl, 0.2 mM EDTA, 0.2 mM EGTA, 0.15 mM spermine, 0.5 mM spermidine with added 1mM DTT and 1 mM PMSF). DNase I digestion with increasing doses of DNase I (Sigma or Roche) was then carried out in DDB supplemented with 1mM CaCl_2_, at 37ºC for 10 minutes, and stopped by the addition of an equal volume of cold stop buffer (50 mM Tris pH 7.5, 150 mM NaCl, 15 mM EDTA, 0.3% SDS). Samples were treated with proteinase K overnight and DNA was extracted with the DNeasy Tissue kit (Qiagen) or phenol/chloroform extraction.

The following primers were used for real-time quantitative PCR, in both DNase I hypersensitivity and ChIP-qPCR assays, in conjunction with SYBR Green PCR Master Mix (Applied Biosystems):

β-actin Forward GTTGGGTGACCCCCAGAAT

β-actin Reverse TTGTGGACACTGCCCCATT

HS1 Forward TTATCTACTCATGAAGGAGCAATGATG

HS1 Reverse GTCAGCTGGGTGGAGTCACA

HS2 Forward GGGTGTGTTCAGCCTTGTGA

HS2 Reverse TCCCTGTGGACTTCCTCCTAGA

HS3 Forward GATGTGTCTATCAGAGGTCCCATATG

HS3 Reverse TCTCCCCACCCTTTGTCCTA

HS4 Forward TGAACAATTTGCCCTCTCTTACATC

HS4 Reverse GGCTTCCTGGTCCAGTAGATAGTATT

Nfm Forward TGCAGGATGAGGTGGCTTTC

Nfm Reverse TGGTGCATGTTCTGGTCTGAGT

**Chromatin Immunoprecipitation (ChIP-qPCR) Assays**

Sorted S0 and S1 cells (at least 10^6^ cells per sample) were cross-linked in 1% formaldehyde for 10 min at room temperature and stopped by adding 150 mM glycine (pH 7). Cells were lysed in immunoprecipitation (IP) buffer (150 mM NaCl, 50 mM Tris-HCl, pH7.5, 5 mM EDTA, 0.5% NP-40, 1% Triton X-100), supplemented with 20 mM sodium butyrate and protease inhibitors (Roche). Chromatin was sonicated 30 times for 10 sec pulses with Misonix Sonicator 3000’s microprobe. For immunoprecipitation, samples were diluted to 500 µl with IP buffer and incubated overnight with antibodies for specific covalent modifications of histones (see list below), followed by a 3-4 hour incubation with Dynabeads protein G (Invitrogen) at 4ºC. Cross-links were reversed at 65ºC overnight in elution buffer (50 mM Tris-HCl, pH 8.0, 10 mM EDTA, 1% SDS). DNA was purified with Zymo Research mini-spin columns; 2 µg herring sperm DNA was used as a carrier.

The following antibodies were used:

Anti H3 acetyl K9, K14, Upstate 06-599

Anti H4 acetyl K5, 8, 12, 16, Upstate 06-866

Anti H3 acetyl K9, Abcam ab4441

Anti H3 dimethyl K4, Abcam ab7766

Anti H3 trimethyl K4, Abcam ab8580

Anti H3 dimethyl K9, Abcam ab1220

Anti H3 trimethyl K27, Active Motif 39535

Anti H3, Abcam ab1791

Anti H4, Abcam ab31827

**DNA Methylation Analysis using Pyrosequencing**

Genomic DNA (gDNA) was isolated using the DNA/RNA Micro Kit (Qiagen). Between 50 and 150 ng of gDNA were treated with sodium bisulfite using the EZ DNA Methylation-Direct Kit (Zymo Research, Orange, CA) to generate bisulfite-converted DNA (bcDNA). Specific primers were designed to the sense strand of the bcDNA sequence, within the HS1 and HS2 sites (see primer sequences below), and used for PCR amplification, with 2 µl of bcDNA as template, in a 30 µl reaction containing 3 µl of 10x PCR buffer (Qiagen), 1.8 µl of 25 mM MgCl_2_, 0.6 µl of 10 mM (each) dNTP, 0.6 µl of 10 µM forward primer, 0.6 µl of 10 µM reverse primer, and 0.15 µl HotStarTaq *Plus* DNA Polymerase (Qiagen). One of the PCR primers was biotinylated to convert the PCR product to single-stranded DNA templates. For the HS1 amplicon, PCR cycling conditions were as follows: 95ºC for 15 min, 45 x (95ºC for 30 sec, 56ºC for 30 sec, 72ºC for 30 sec), then 72ºC for 5 min. The HS2 amplicon used 61ºC for annealing.

Calculation of CpG methylation and verification of complete bisulfite conversion of template DNA was performed on the PCR product by EpigenDx (Worcester, MA) using the Pyrosequencing PSQ96 HS System (Qiagen) as per manufacturer’s instructions. Pyrosequencing for allele quantification is a real-time, sequencing-based DNA analysis that quantifies multiple, and consecutive CpG sites individually.

HS1 primers:

Forward: GGTATATTTGAAAATTTTGGTAATAGT

Reverse: CAACAAATAAAAACCAAAACTACACA

HS2 primers:

Forward: GGTTTTTTGGTAGATAGGTTATATGTG

Reverse: AAAACTAAACACACCCACAAAATA

**Cytospin preparation**

Fetal liver cells (freshly sorted, or after retroviral infection and incubation in Epo for 24-48 hours) were cytospun onto coated glass slides at 800 rpm for 5 minutes (Single Cytofunnel, Fisher Brand). The slides were air-dried, methanol fixed, stained with diaminobenzidine hydrochloride followed by Wright-Giemsa stain (Sigma). Digital images were taken with a Zeiss microscope using a SPOT Flex Camera (Diagnostic Instruments, Inc) and analyzed with version 4.5 Spot imaging software.

**Single cell PCR analysis**

S0 cells were individually sorted from fresh fetal liver directly into 96 well plates containing lysis buffer (0.4% NP-40, 25 µM DTT, 0.5 U RNase Out and 65 µM dNTPs). Reverse transcription was performed directly on cell lysates according to the manufacturer’s instructions (Qiagen Sensiscript RT kit or Invitrogen SuperScript III First Strand Synthesis System kit). EpoR and β-actin were amplified from 2 µL of cDNA via nested PCR. Each experiment included spleen cDNA (positive control), thigh muscle cDNA and no template (negative controls)

The following primers were used:

β-actin Forward: CTA GGC ACC AGG GTG TGA TGG

β-actin Reverse: TCT CTT TGA TGT CAC GCA CGA

EpoR Forward: aac ttc cag ctg tgg ctg ctg

EpoR Reverse: cct tgt ccc aga ctc aga g

Nested PCR:

β-actin Forward: CGA GGC CCA GAG CAA GAG AG

β-actin Reverse: CGG TTG GCC TTA GGG TTC AG

EpoR Forward: cct act tgg tat tgg

EpoR Reverse: cct cac ctt cca gct ttg ag

**FISH**

Cell preparation: BrdU pulse labeling was done in vivo by injecting pregnant females with BrdU intraperitoneally. Sorted S0 and S1 (0.5 to 1 x 10^6^ cells for each) were washed twice in 5 ml cold PBS/2% sucrose, and fixed by adding 5 ml of cold Methanol/Acetic acid mixture (3:1) dropwise, while vortexing. Cells were fixed for 10 min at 4ºC, washed x3 in Methanol/ Acetic acid and stored at -20ºC until used for FISH. Alternatively, sorted S0 and S1 cells were suspended at 50,000 cells in 200 ml PBS/2% sucrose and cytospins prepared by spinning 5 min at 800 rpm, allowed to dry briefly (10-20 seconds), and fixed in a 50 ml Falcon containing 20 ml Methanol/Acetic acid at 4ºC for 10 min; slides were poly-L lysine coated.

Prior to FISH, fixed cells on slides were treated in 70% ethanol, subjected to pepsin digestion (0.01%) and post-fixed in 3.7% paraformaldehyde. Cells were dehydrated in successive 3 min washes of 70%, 90% and 100% ethanol and air-dried and used for probe hybridization on the same day.

Probe to the β-globin major gene was labeled with digoxigenin and resuspended in hybridization mix in the presence of tRNA and mouse COT DNA, used at 20 ng/microliter, 50 microliters per slide. Hybridization was carried out in a humidified chamber at 37% overnight in hybridization mix containing 10 mg/ml sonicated Salmon Sperm DNA, 50% formamide, 2X SSC, 50 mM sodium phosphate buffer and 10% dextran sulphate. Immunostaining was performed by labeling slides with sheep anti-digoxigenin (Roche, 1 333 089) followed by fluorescein-conjugated rabbit anti-sheep IgG (402104, EMD Bioscience, NJ, USA) and then FITC-conjugated goat anti-rabbit IgG together with mouse anti-BrdU IgG (M0744, Dako, CA, USA) followed by Texas Red-conjugated donkey anti-mouse IgG (Jackson immunoresearch). Nuclei were counterstained in DAPI and visualized using fluorescence microscopy.

**Quantitative Western blot analysis**

Sorted cells from each fetal liver subset were lysed by rocking for 15 min at 4°C in lysis buffer (1% NP40, 50 mM Tris pH 7.4, 150 mM NaCl, 1 mM EDTA, 10% glycerol supplemented with protease inhibitors); following centrifugation for 15 min at 4°C, supernatant was quantified by the BCA Protein Assay Kit (Pierce). Lysates were analyzed on a 4-12% gradient polyacrylamide gel (Invitrogen), transferred to a nitrocellulose membrane, and probed with Rabbit polyclonal to beta actin (ab8227, Abcam), and with Mouse monoclonal to p57^KIP2^ (sc-56341, Santa Cruz) overnight at 4ºC. Membranes were probed simultaneously with anti-Rabbit IRDye 680 (red channel) and anti-Mouse-IRDye 800CW (green channel) and analyzed for both β-actin and p57^KIP2^ using the Odyssey infrared imaging system (LI-COR Biosciences). Target protein bands were quantified using the Odyssey software v3 (LI-COR).
